# Quantitative proteomics of small numbers of closely-related cells: Selection of the optimal method for a clinical setting

**DOI:** 10.3389/fmed.2022.997305

**Published:** 2022-09-27

**Authors:** Kyra van der Pan, Sara Kassem, Indu Khatri, Arnoud H. de Ru, George M. C. Janssen, Rayman T. N. Tjokrodirijo, Fadi al Makindji, Eftychia Stavrakaki, Anniek L. de Jager, Brigitta A. E. Naber, Inge F. de Laat, Alesha Louis, Wouter B. L. van den Bossche, Lisette B. Vogelezang, Rutger K. Balvers, Martine L. M. Lamfers, Peter A. van Veelen, Alberto Orfao, Jacques J. M. van Dongen, Cristina Teodosio, Paula Díez

**Affiliations:** ^1^Department of Immunology, Leiden University Medical Center (LUMC), Leiden, Netherlands; ^2^Leiden Computational Biology Center, LUMC, Leiden, Netherlands; ^3^Center for Proteomics and Metabolomics, LUMC, Leiden, Netherlands; ^4^Department of Neurosurgery, Erasmus MC, Rotterdam, Netherlands; ^5^Translational and Clinical Research Program, Cancer Research Center (IBMCC; University of Salamanca-CSIC), Salamanca, Spain; ^6^Cytometry Service, NUCLEUS, Department of Medicine, University of Salamanca and Institute of Biomedical Research of Salamanca (IBSAL), Salamanca, Spain

**Keywords:** proteome characterization, low cell numbers, closely-related cells, monocyte, macrophage, paucicellular clinical samples

## Abstract

Mass spectrometry (MS)-based proteomics profiling has undoubtedly increased the knowledge about cellular processes and functions. However, its applicability for paucicellular sample analyses is currently limited. Although new approaches have been developed for single-cell studies, most of them have not (yet) been standardized and/or require highly specific (often home-built) devices, thereby limiting their broad implementation, particularly in non-specialized settings. To select an optimal MS-oriented proteomics approach applicable in translational research and clinical settings, we assessed 10 different sample preparation procedures in paucicellular samples of closely-related cell types. Particularly, five cell lysis protocols using different chemistries and mechanical forces were combined with two sample clean-up techniques (C18 filter- and SP3-based), followed by tandem mass tag (TMT)-based protein quantification. The evaluation was structured in three phases: first, cell lines from hematopoietic (THP-1) and non-hematopoietic (HT-29) origins were used to test the approaches showing the combination of a urea-based lysis buffer with the SP3 bead-based clean-up system as the best performer. Parameters such as reproducibility, accessibility, spatial distribution, ease of use, processing time and cost were considered. In the second phase, the performance of the method was tested on maturation-related cell populations: three different monocyte subsets from peripheral blood and, for the first time, macrophages/microglia (MAC) from glioblastoma samples, together with T cells from both tissues. The analysis of 50,000 cells down to only 2,500 cells revealed different protein expression profiles associated with the distinct cell populations. Accordingly, a closer relationship was observed between non-classical monocytes and MAC, with the latter showing the co-expression of M1 and M2 macrophage markers, although pro-tumoral and anti-inflammatory proteins were more represented. In the third phase, the results were validated by high-end spectral flow cytometry on paired monocyte/MAC samples to further determine the sensitivity of the MS approach selected. Finally, the feasibility of the method was proven in 194 additional samples corresponding to 38 different cell types, including cells from different tissue origins, cellular lineages, maturation stages and stimuli. In summary, we selected a reproducible, easy-to-implement sample preparation method for MS-based proteomic characterization of paucicellular samples, also applicable in the setting of functionally closely-related cell populations.

## Introduction

In the era of high-throughput cell analysis, techniques such as RNA sequencing, flow cytometry (FC) and mass cytometry have led to important scientific advances, not only in basic research, but also in translational and clinical research settings (1–4). Despite their growing role, these methods have several limitations, e.g., RNA profiles do not necessarily reflect the protein expression patterns and cytometry-based approaches only evaluate a restricted number of pre-selected proteins (up to 60), thereby providing limited and biased information on the cell's proteome (5, 6). Mass spectrometry (MS) has the potential to overcome these handicaps. In fact, MS-based studies have improved the knowledge of cellular mechanisms and functions in e.g., rheumatoid arthritis (7), diverse cancer types (8–10) and dementia (11). However, translational and clinical research studies are often challenged by limited sample availability, such as scarce patient material and small target populations (e.g., tumor-infiltrating immune cell populations). Moreover, investigations on closely-related cells, such as different maturational stages within a population (e.g., in hemato-oncological disorders like myelodysplastic syndromes), could be difficult to perform since unique identities of small cell subsets can be due to subtle differential protein expression levels, which might be ultimately complex to assess in paucicellular specimens (12, 13). Furthermore, in translational research and clinical studies, the evaluation of large sets of samples is often required to obtain reliable conclusions for future application in patient care, requiring rapid, cost-effective and reproducible techniques.

While there have been advances in MS-based applications in e.g., cancer research, standardized MS strategies for proteome characterization of small cell populations are still lacking (12, 13). In fact, even though innovative approaches for single-cell proteomics have been recently developed (14–18), their application on a routine basis in a clinical setting is not (yet) feasible. The required platforms are still in development and need specific devices (e.g., microfluidic chips, liquid handling systems), which are usually home-built, expensive, and time-consuming, and include complex non-standardized protocols which demand for extensive proteomics knowledge. Therefore, to promote the use of MS-based techniques for analysis of limited cell numbers in non-specialized proteomics environments, and to improve its broader applicability, it is necessary to select optimal approach(es) in terms of performance, reproducibility, accessibility, throughput, ease of use, and cost and time effectiveness.

In this regard, the definition of a simple but efficient sample preparation protocol for MS studies, only requiring routine laboratory equipment but allowing for highly reliable data, could lead to a more prominent role of MS-based proteomics in translational research with the potential of application in patient care. The plethora of protocols available in the literature might be too overwhelming for researchers who are not specialized in MS-based proteomics. For instance, multiple different approaches are provided for cell lysis, including the usage of different components (e.g., chaotropes as urea or detergents as SDS) and conditions (e.g., ultrasonication, heat shock), which finally determine the efficiency of protein extraction (19). Likewise, removal of detergents, salts and/or other contaminants present in the sample can be performed in several manners, using traditional strategies such as gel electrophoresis (20) or C18 StageTips (21), or newer methods such as Filter-Aided Sample Preparation (FASP) (22) or single-pot, solid phase-enhanced sample preparation (SP3) (23, 24).

Altogether, there is a need for systematic evaluation of the performance, accessibility and user-friendliness of sample preparation methods for MS-based analysis for application in non-specialized settings where proteome investigations might be highly valuable, particularly for paucicellular studies. To this end, we selected five lysis procedures based on their different chemistries (chaotrope, detergent, hypotonic buffer, cosolvent) and physical-mechanical forces (ultrasonication, heating, thawing/freezing) representing the most popular proven methods. Also, SP3 and C18 were chosen as an example of new, highly effective vs. traditional clean-up approaches. All possible lysis method-clean-up approach combinations were then evaluated to select the optimal procedure for complete quantitative proteome profiling of low numbers of closely-related cells (from 50,000 cells down to 2,500 cells), with application in translational/clinical settings. Cells from different origin (non-hematopoietic vs. hematopoietic) and different maturational stages (monocytic cell populations) were used as model systems. This comparative study was structured in three phases: (i) cell lines for monocytes, macrophages and colon adenocarcinoma cells (as non-hematopoietic cell model) were employed to define the best strategy, (ii) small cell numbers of the three major monocyte subsets from PB and macrophages/microglia (MAC) from glioblastoma (GBM) patients (together with T cells from both tissues) were investigated in depth, and (iii) validation of the performance of the method was done by using high-end FC and its feasibility was proven in 38 different cell types from five human tissues. Results confirmed the usefulness of the selected method to define the proteome landscapes and maturational relationships of the analyzed cell subsets, being affordable and easy to implement in a translational/clinical laboratory.

## Materials and methods

### Cell culture

The THP1 acute monocytic leukemia cell line (DSMZ ACC 16) was selected as a model for monoblasts and macrophages (after *in vitro* differentiation, dTHP1), whereas the HT-29 human colon adenocarcinoma cell line (DSMZ ACC 299) was chosen as a non-hematopoietic lineage tumor model. THP1 and HT-29 cells were cultured in RPMI 1640 (Lonza, Basel, Switzerland) and McCoy's medium (Sigma-Aldrich, St. Louis, MO/US), respectively. Both media were supplemented with 10% heat-inactivated fetal calf serum (Sigma-Aldrich), 100 U/mL penicillin, 100 μg/mL streptomycin and 1% GlutaMAX™ Supplement (Gibco, Gaithersburg, MD/US). To induce differentiation into macrophages, THP1 cells were treated with 200 nM phorbol 12-myristate 13-acetate (PMA; Sigma-Aldrich) for 72 h and rested in a PMA-free medium for 5 days as described by Daigneault et al. (25). All cell cultures were maintained at 37°C in a humidified atmosphere and 5% CO_2_.

### Human sample collection

A total of 6 fresh ethylenediamine tetraacetic acid (EDTA)-anticoagulated human PB samples from healthy donors (4:2 female: male ratio, median age 33.5, range 27–40) and 5 tumor tissue samples from GBM patients (2:3 female: male ratio, median age 60, range 46–70) were collected after written informed consent was given by each donor and/or his/her legal representative(s) according to the Declaration of Helsinki, guidelines of the local ethics committees and review boards (PB: LUMC Volunteer Donor Service, B18.031, project request L18.001; GBM: Medical Ethical Committees of Erasmus Medical Center Rotterdam, 2013-139). The processing of PB and GBM samples is described in Supplementary Experimental Procedures.

### Sample processing for cell population sorting

Three major monocyte subsets (i.e., CD14^++^, CD16^−^ classical monocytes, cMo; CD14^++^, CD16^+^ intermediate monocytes, iMo; and CD14^−/*dim*^ CD16^hi^ non-classical monocytes, ncMo) and T cells from PB, and MAC and T cells from GBM samples were sorted (Supplementary Figure 1) with a purity systematically higher than 98.2% ± 3.3% employing a 4-way fluorescence-activated cell-sorter (FACSAria; Becton Dickinson Biosciences – BDB – San Jose, CA, US), equipped with the FACSDiva software (BDB). Before sorting, samples were stained using a stain-wash procedure. Shortly, PB mononuclear cells (PBMCs) and GBM cells were stained with distinct 7-color monoclonal antibody panels (Panels A and B, Supplementary Table 1), incubated rolling for 30 min at room temperature (RT) in the dark and washed with phosphate-buffered saline (PBS). GBM cells were further incubated in 1:1000 viability marker (LIVE/DEAD™ Fixable Aqua Dead Cell Stain Kit, Thermo Fisher, Waltham, MA/USA) for 30 min at RT protected from light, following the manufacturer's instructions. Finally, both PBMCs and GBM cells were washed and resuspended in PBS for subsequent sorting. All cell populations were sorted at 4°C and collected in RPMI supplemented with 10% fetal calf serum and 1% protease/phosphatase inhibitor cocktails (Sigma-Aldrich) to preserve cell viability and protein integrity, respectively. Samples were washed three times with ice-cold PBS supplemented with 1% protease/phosphatase inhibitor cocktails (5 min, 1,000 *g*, 4°C) and cell pellets were freeze-dried and stored at −80°C until further processing.

### Sample processing strategies for MS/MS proteomics analysis

**Figure 1:**
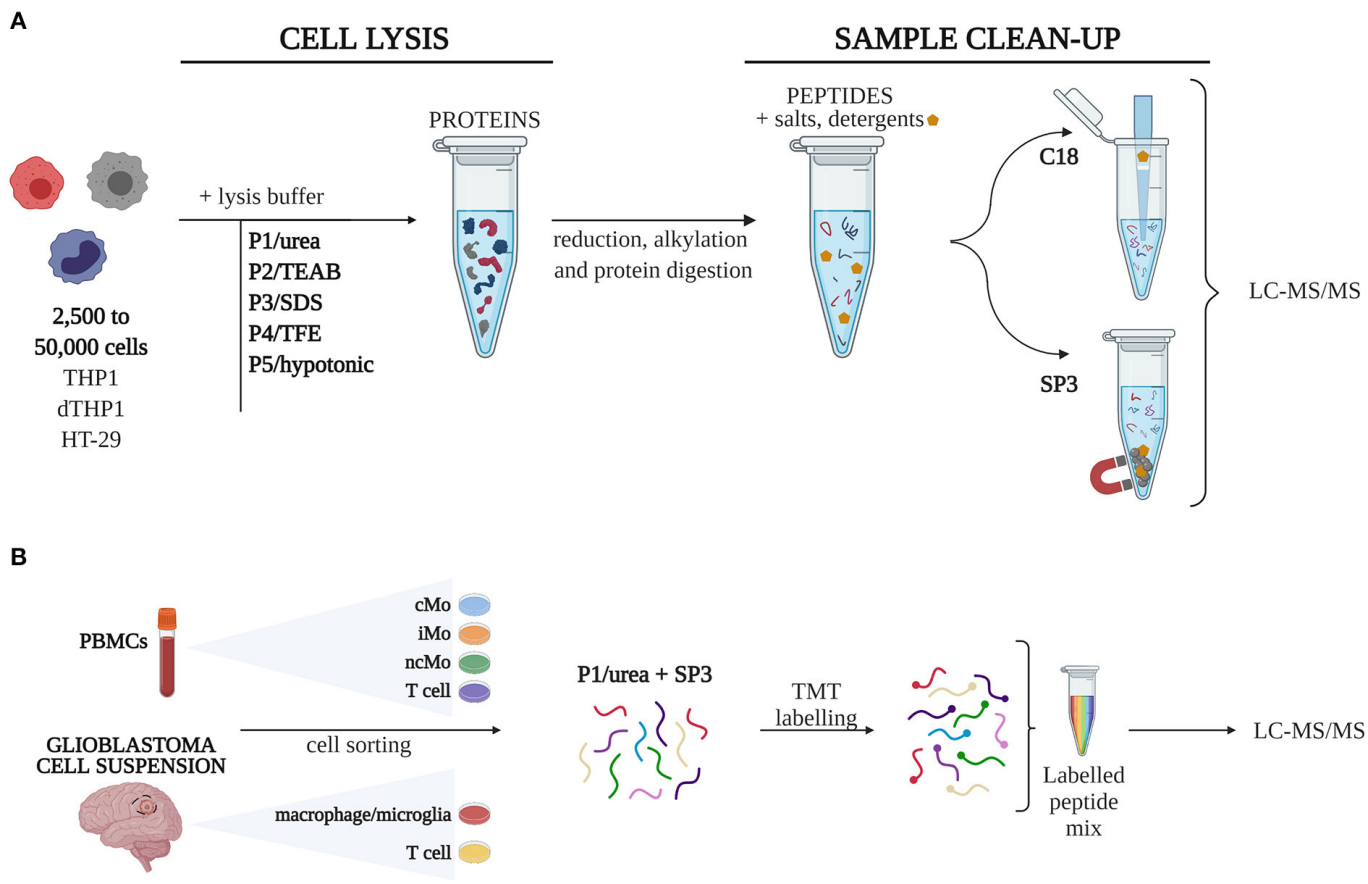
Experimental design. **(A)** Five different cell lysis buffers were combined with C18 and SP3 clean-up approaches to define 10 procedures, which were tested for their performance on quantity-limited samples (2,500 to 50,000 cells). **(B)** The combination of urea-based lysis buffer and SP3 was applied to study different monocytic subsets isolated from peripheral blood mononuclear cells (PBMCs) and macrophages from glioblastoma. Samples were labelled with TMT16-plex tags for quantitative analysis. *cMo*, classical monocytes; *iMo*, intermediate monocytes; *LC-MS/MS*, liquid chromatography-tandem mass spectrometry; *ncMo*, non*-*classical monocytes; *SDS*, sodium dodecyl sulfate; *SP3*, single-pot, solid-phase-enhanced sample preparation method; *TEAB*, triethylammonium bicarbonate; *TFE*, 2,2,2-trifluoroethanol; *TMT*, tandem mass tag [Created with BioRender.com].

A total of 10 sample processing strategies, combining five different cell lysis procedures (P1-5) with two well-described approaches for sample clean-up and peptide recovery (SP3, C18), were evaluated in triplicates in 50,000 (50k), 10,000 (10k), and 2,500 (2.5k) cells (Figure 1). 10^6^ cells were used as a reference (Ref) sample of maximum proteome coverage.

### Protein extraction procedures

Lysis buffer volume per cell number was determined by titration (data not shown) to achieve maximum protein extraction and minimum sample dilution. Thus, 40, 50, 70, and 100 μL of lysis buffer were used for 2.5, 10, 50k, and 10^6^ cells, respectively. After lysis, samples were stored at −80°C until further processing.

- **P1: Urea-based lysis buffer** [adapted from (26)]. Cell pellet was lysed in 20 mM HEPES pH 8.5, 9 M urea, 1 mM sodium orthovanadate, 2.5 mM sodium pyrophosphate and 1 mM ß-glycerophosphate, followed by sonication in an ice-water bath (3 cycles, 5 s each) and centrifugation at 21,000 *g* at RT for 15 min. The supernatant containing the proteins was stored.
- **P2: Triethylammonium bicarbonate (TEAB)-based lysis buffer** [as described by (27)]. Briefly, cells were lysed in TEAB lysis buffer (8 M urea in 100 mM TEAB pH 8.5) followed by freeze-drying and overnight storage at −80°C. Next day, samples were sonicated in an ice-water bath (30 cycles, 30 s on/30 s off) and stored.
- **P3: Sodium dodecyl sulfate (SDS)-based lysis buffer** [adapted from (23)]. A lysis buffer containing 0.5% SDS, 50 mM HEPES pH 8.5, 1 μg/μL DNase and 1% protease/phosphatase inhibitor cocktails was used for protein extraction. The mixture was heated (5 min, 95°C) and centrifuged (21,130 *g*, 15 min, RT) to collect the supernatant.
- **P4: Trifluoroethanol** (**TFE)-based lysis buffer** [as described by (28)]. Briefly, cells were lysed by adding a TFE lysis buffer (50% TFE in 50 mM ammonium bicarbonate pH 8.0) and incubated at 60°C for 2 h, before sonication in an ice-water bath for 2 min.
- **P5: Hypotonic lysis buffer** (method developed *in-house* based on previous knowledge, more information in Supplementary materials and methods). Cell lysis was performed in 30 mM HEPES, 0.5 mM dithiothreitol (DTT), 0.1 mM EDTA, and 1% protease/phosphatase inhibitor cocktail. The sample was incubated at 4°C for 5 min before undergoing four freeze-thaw cycles in dry-ice and 42°C, respectively. The supernatant containing the proteins was collected after centrifugation (21,130 *g*, 15 min, 4°C).

All samples were quantified and silver**-**stained as described in Supplementary materials and methods.

### Sample clean-up and peptide recovery

Proteins were reduced with DTT and alkylated with iodoacetamide before protein digestion was carried out with Lys-C (4 h) and/or trypsin (overnight), applying the conditions described by Sielaff et al. (26), Bensaddek et al. (27), Hughes et al. (23), and Wang et al. (28) for samples lysed with methods P1, P2, P3, and P4, respectively. For P5-lysed samples, conditions were defined based on previous experience and published optimizations (29). More details in Supplementary materials and methods. Subsequently, peptide samples were subjected to clean-up and recovery approaches (i.e., SP3 and/or C18) for further liquid chromatography (LC)-MS/MS analysis.

- **SP3 technology**. In this single-pot, solid-phase-enhanced sample preparation method described by Hughes et al. (23) and modified by Sielaff et al. (26) and Hughes et al. (24), protein digestion and peptide purification are performed on beads (Sera-Mag Carboxylate-Modified Magnetic Particles; GE Healthcare, Chicago, IL/US). Briefly, protein samples were incubated twice with beads in 70% acetonitrile (ACN) for 18 min. After incubation, bead-bound proteins were retained by a DynaMag™-PCR Magnet (Thermo Fisher) and the supernatant containing detergents, salts and other contaminants was discarded. After three washing steps with 70% ethanol and ACN, bead-bound proteins were enzymatically digested. Afterwards, peptides were eluted with 2% DMSO, lyophilized in a freeze dryer and stored at −20°C.
- **C18 column-based approach**. This method only concerns the peptide clean-up and recovery, as protein digestion must be performed before in a tube. C18 StageTips were prepared by driving a needle through an Empore™ C18 Extraction Disk and inserting the membrane cut-out inside a tip, as described by Rappsilber et al. (30). Columns were activated with 100% methanol, 50% ACN and 0.1% trifluoroacetic acid (TFA). Acidified peptide samples were loaded onto the column and after washing with 0.1% TFA, peptides were eluted with 0.1% TFA/70% ACN, lyophilized and stored at −20°C.

### Tandem mass tag (TMT) labeling

Lyophilized peptides from 2.5 and 50k cMo, ncMo, iMo and T cells from PB and MAC and T cells from GBM samples were directly labeled for 1 h at RT with 10 and 20 μg of TMT reagents (TMTpro™ 16plex experiment, Thermo Scientific), respectively, after resuspension in 40 mM HEPES pH 8.4. The reaction was quenched with 5% hydroxylamine for 15 min. TMTpro labeled samples were randomly pooled in 5 different TMT sets (each containing 54 μg protein) and lyophilized. A mix of PBMC and GBM proteins was used as a bridge sample (TMTpro-134N tag) to normalize the data across TMT sets.

### MS/MS data analysis

Sample processing for LC-MS/MS analysis is described in Supplementary materials and methods.

Raw data files from the technical evaluation analysis with cell lines were converted to mgf using the msConvert tool (ProteoWizard toolkit, v3.0.20157) (31). Peak lists obtained from MS/MS spectra were identified using X!Tandem Vengeance v2015.12.15.2 on SearchGUI v3.3.5 (32). Protein identification was conducted on PeptideShaker v1.16.31 (33) against a concatenated target/decoy UniProtKB database [release Oct 2018, 42,517 (target) sequences], including the cRAP database (common Repository of Adventitious Proteins, v2012.01.01; The Global Proteome Machine). Search parameters were set as follows: fully tryptic digestion (no P rule) with up to 3 missed cleavages; 10 ppm and 0.02 Da as the precursor and fragment mass tolerances, respectively; and carbamidomethylation of Cys and acetylation of protein N-term and oxidation of Met as fixed and variable modifications, respectively.

For TMT analysis on PB and GBM cell populations, raw data were first converted to peak lists using Proteome Discoverer v2.4 (Thermo Electron) and submitted to the UniProtKB database (*Homo sapiens minimal*, 20,596 entries), using Mascot v2.2.07 for protein identification. Search parameters were set as follows: 10 ppm and 0.02 Da as the precursor and fragment mass tolerances, respectively; fully tryptic digestion (no P rule) with up to 2 missed cleavages; oxidation of Met was set as variable modification and carbamidomethylation of Cys, and TMT16plex on N-term and Lys were set as fixed modifications. The 5 TMT-16plex analyses were normalized to each other by the bridge sample.

In both data analyses, a 1% false discovery rate (FDR) was set for peptide spectrum matches (PSM), peptides and proteins. Moreover, only proteins with at least 2 unique peptides identified in all technical and/or biological replicates were considered in the analysis, unless otherwise indicated.

The MS data along with the identification results have been deposited to the ProteomeXchange Consortium (34) via the PRIDE partner repository (35) with the dataset identifiers PXD018872 (cell lines) and PXD026604 (PB and GBM).

### Sample processing for immunophenotypic studies

Two FC combinations of 17 and 13 fluorochrome-conjugated antibodies (Panels C and D, Supplementary Table 1) were designed to validate the MS-based characterization performed on both sorted PB and GBM subpopulations. Markers were selected based on their high protein coverage by MS and their varying expression levels across subsets. Proteins including isoforms were not considered. Both antibody panels C and D included a common backbone of 6 markers for population immunophenotypic identification (i.e., CD45 for leukocytes, CD33 for myeloid cells, HLA-DR for total antigen-presenting cells (APC), CD14 and CD16 for monocyte subsetting and CD64 for microglia/macrophages). These 6 markers were also combined in panel E (Supplementary Table 1) to determine background staining and determine presence/absence of a protein.

Samples were processed according to the sample preparation and staining standard procedures described at www.EuroFlow.org. All incubations were performed at RT in the dark. Briefly, a total of three tubes per sample (containing 10 × 10^6^ PBMCs or 0.5 × 10^6^ GBM cells/tube) were stained with antibody panels C, D, and E, respectively, for 30 min, washed with PBS and incubated with a viability marker (Zombie NIR, Biolegend, San Diego, CA/USA) for 30 min. Cells were washed once and subsequently incubated with 100 μL of reagent A of the Fix&Perm™ Cell Permeabilization Kit (Sanbio, Uden, The Netherlands) for 15 min. After a washing step with PBS containing 0.5% bovine serum albumin, 0.1% sodium azide and 2 mM EDTA (pH 7.4), cells were resuspended in 100 μL of reagent B (Fix&Perm™ Cell Permeabilization Kit) and incubated again with antibody panels C and D for 15 min to allow intracellular staining. Finally, cells were washed once and resuspended in 500 μL of PBS for acquisition using a three laser (405, 488, 640 nm) Aurora spectral flow cytometer (Cytek Biosciences, Fremont, CA, US). Data analysis was performed with Infinicyt™ software v2.0.2.d.000 (Cytognos S.L., Salamanca, Spain).

### Ethics approval

All procedures were performed in accordance with the ethical standards of the responsible committee on human experimentation (institutional and national) and with the Helsinki Declaration of 1964, as revised in 2013. Informed consent was obtained from all patients included in the study.

### Experimental design and statistical rationale

For all MS-based experiments with cell lines, three technical replicates were used. MS-based and FC experiments with biological samples were performed with at least 5 donors. Biological samples collected for MS experiments were labeled with TMT separately and combined. Additional details can be found in the text above. For continuous variables, mean and standard deviation (SD) were calculated. For non-continuous variables, median and range were determined and statistical significance (*p*-value < 0.05) was calculated by the non-parametric Kruskal-Wallis and Mann-Whitney U tests with Dunns' and Bonferroni's test, respectively, to correct for multiple comparisons. The degree of correlation between two different cell conditions was determined by the Spearman correlation. All statistical analyses were performed using GraphPad Prism 8.0 software (GraphPad Prism, San Diego, CA, USA). Proteome Discoverer v2.4 (Thermo Fisher) was employed for principal component analysis (PCA), hierarchical clustering analysis and Volcano plots.

## Results

### Performance of cell lysis procedures combined with SP3 and C18 sample clean-up methods

**Figure 2:**
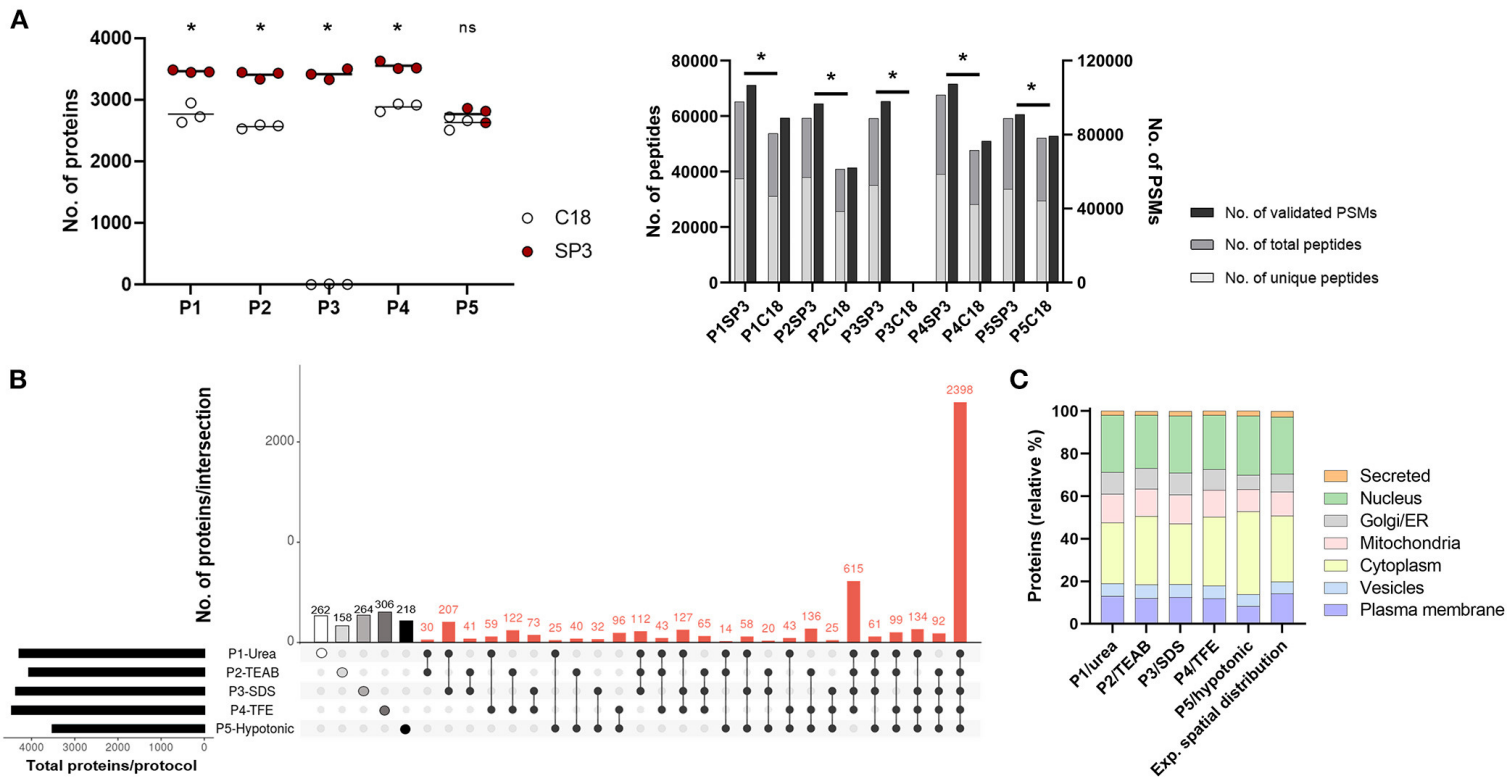
Performance of P1-P5 cell lysis protocols combined with SP3 or C18 clean-up methods in 50,000 dTHP1 cells. Experiments were run in triplicate and depicted proteins were identified with at least 2 unique peptides. **(A)** Number of proteins (left panel), peptides (total and unique) and validated peptide spectrum matches (PSMs) (right panel) identified per procedure. **(B)** Attribute plot displaying the qualitative proteomic analysis of P1–P5 combined with SP3. Here, maximum proteome coverage (i.e., all proteins identified by any of the three replicates per protocol) was considered in the analysis. Each vertical bar shows unique protein numbers and corresponds to either a unique procedure (white-, light grey-, medium grey-, dark grey- and black-filled dots for P1-, P2-, P3-, P4-, and P5-SP3 combinations, respectively) or a set of procedures (black dots interconnected by lines). The bar chart on the bottom left side plots the total number of proteins identified per protocol. **(C)** Subcellular location distribution of proteins identified per protocol and expected spatial distribution for dTHP1 proteome (based on all protein datasets for this cell model). Protein numbers are expressed in relative percentages (%). The main subcellular location per protein was assigned according to UniProt database. Data used for the graph is collected in Supplementary Table 5. *P1*, urea-; *P2*, TEAB-; *P3*, SDS-; *P4*, TFE-; and *P5*, hypotonic-based cell lysis. *p-value <0.05 and 10% FDR; *ns*, not significant. Significant differences were calculated using the Mann Whitney test.

Firstly, we assessed five cell lysis protocols (P1–P5) in THP1, dTHP1 and HT-29 cell lines by determining the extracted protein amount (Supplementary Figure 2). Quantification of 50k cells revealed that P5/hypotonic was the least efficient method (in dTHP1 and HT-29 cells), whereas P3/SDS reported the highest protein yield in the three cell lines. Also, silver staining of P1-to-P5-lysed samples (Supplementary Figure 3) showed highly similar protein distribution profiles for the same cell types. Since enough protein was obtained in all cases, only dTHP1 cells were used to evaluate the performance of all lysis methods combined with SP3 and C18 clean-up strategies to reduce the technical complexity of the study. For the same purpose, we compared two different LC gradient times (60 and 160 min) (Supplementary Figure 4 and Supplementary Tables 2, 3) in five randomly selected protocol combinations seeking to reduce the total LC-MS/MS measurement time. A significant improvement (2- to 10-fold; *p*-value < 0.05) in protein identification was observed when using the longer gradient. The analysis of the SP3- vs. C18-combined procedures (Figure 2A and Supplementary Table 3) reported better performance of the bead-based system, with significant differences in terms of identified proteins, peptides and PSMs between both sample clean-up strategies when coupled to P1–P4. Although the same pattern was shown for peptides and PSMs for P5-SP3 vs. P5-C18, total protein identifications did not significantly differ between the clean-up approaches. Of note, P3-C18 only revealed a dozen of proteins. Finally, processing cost and time and throughput parameters were also evaluated (Supplementary Tables 4A,B) indicating comparable pricing for both alternatives and a shorter processing time for the C18 method vs. SP3 when only assaying one sample at a time (10–30 min vs. >60 min). However, the throughput of the bead-based system is superior leading to higher time efficiency. Based on these results, only SP3-coupled methods were further assessed.

### Selection of the best lysis protocol-SP3 combination for the analysis of small clinical samples

Overall, SP3-combinations performed similarly concerning the total proteins identified regardless the P-cell lysis used (Figure 2A), except for P5 (~1,000 fewer proteins detected). On average, ~3,500 proteins (with ≥2 unique peptides), 48,000 peptides and 77,000 PSMs were identified per protocol for dTHP1 cells, with a total of 5,975 distinct proteins detected throughout the five procedures. When considering the maximum proteome coverage by protocol (i.e., all proteins identified in any of the replicated experiments), an overlap of 40% (2,398/5,975) proteins among the five combinations was observed (Figure 2B), and one-fifth (1,208/5,975) of all proteins were exclusively identified by any of the five procedures (Supplementary Table 5), with P1-, P3- and P4-SP3 showing the highest recoveries (262, 264, and 306 unique proteins, respectively).

To further select SP3 combinations for subsequent evaluation in small inputs, and in addition to proteome coverage, several parameters were considered: *(i)* technical reproducibility, *(ii)* spatial distribution, *(iii)* functional annotation per protocol, *(iv)* processing time, *(v)* cost, *(vi)* accessibility, and *(vii)* ease of use (Supplementary Table 4). *(i)* To assess the data quality and the reproducibility of the protocols, correlations were calculated for the technical replicates using the relative normalized spectral abundance factor (NSAF) values (Supplementary Figure 5A). P1-, P3- and P4-SP3 procedures were the best performers, whereas P2-SP3 showed poor correlations for 2 out of 3 replicates (r^2^ = 0,44). *(ii)* As for the spatial distribution analysis (Figure 2C), the expected proportion of proteins per subcellular location (according to the database) was used as a reference to determine which method better reflects the spatial composition. Deviation vs. the reference distribution was calculated for each subcellular compartment (Supplementary Table 5). Overall, the P1/urea method better reflected the actual protein distribution across subcellular locations (approach showing the lowest variation, average: 12.8%), closely followed by P4/TFE and P3/SDS, and with P5/hypotonic depicting the highest variation (average: 17.6%). When taking a closer look at the data, it was observed that the cell membrane and nucleus compartments were better represented by P1 compared to other methods and that there was no compartment where it performed the worst. P2/TEAB was better at isolating cytoplasmic proteins (as P4 also was), whereas P3 worked well for the nuclear subfraction. *(iii)* Extracted proteins were annotated to their function using the Reactome pathway database (36). Overall, no significant differences were observed between the protocols (Supplementary Figure 6A and Supplementary Table 6), with most of the pathways less represented by proteins identified by P5-SP3. *(iv)* Average processing time was 19 h, with P3 being the shortest (caused by elimination of the Lys-C digestion step) and P2 the longest (almost 30 h). *(v)* Pricing varied from €1.60/sample for P3 and P5 to €2.60 for P2 and P4 methods. P1 had an intermediate cost of €2.04 per sample. *(vi)* All tested protocols were accessible and *(vii)* easy to use, however, P2 was the most tedious due to the 30 cycles of on/off sonication (if using a non-programmable ultrasonic bath).

Based on the lower total number of proteins, peptides and PSMs identified, P5-SP3 was excluded for further analysis. Since the assessment of the technical reproducibility reported poor correlations for P2-SP3, this combination was also discarded. As for the other three approaches, the data quality evaluation (Supplementary Figure 5A) reported a better correlation between P1–P3 than P1–P4 and P3–P4. Aiming at selecting the best combination, further testing of P1/urea- and P3/SDS-SP3 strategies on other cell models (i.e., THP1 and HT-29) (Supplementary Figure 7 and Supplementary Table 7) was performed. Overall, no significant differences were observed between the two approaches. Hence, considering the preference for non-detergent based methods for downstream steps (due to potential issues of detergents during MS analysis), and considering the high number of proteins, peptides and PSMs obtained, the greater reproducibility, the unbiased representation of proteins isolated from the different cell compartments (with a very good performance for membrane and nuclear proteins), its ease of use, required processing time, affordable cost and required equipment, P1/urea-SP3 was selected as the optimal procedure to investigate the proteome of low numbers of closely-related cells.

### Performance of P1/urea-SP3 procedure in low cell numbers

**Figure 3:**
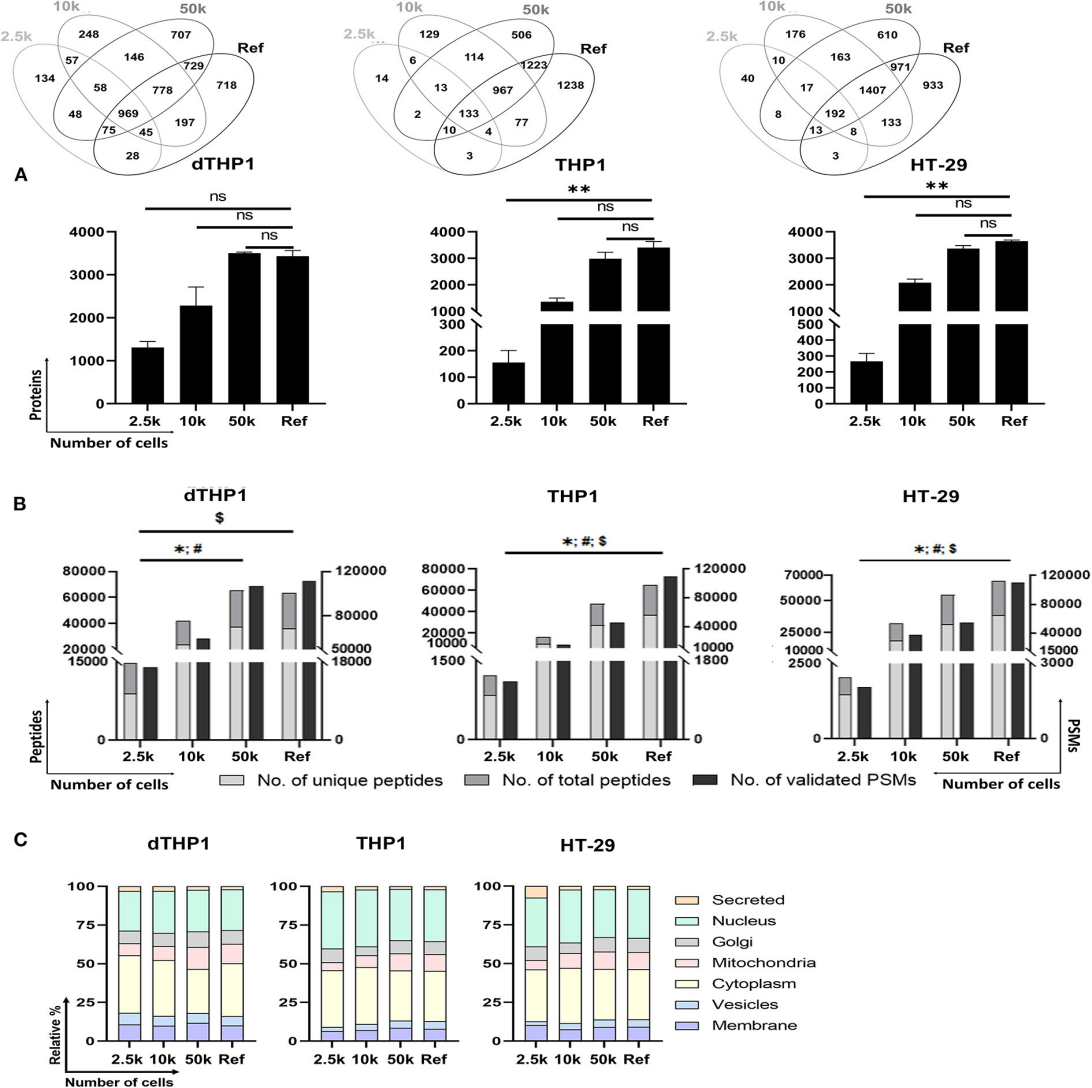
Performance of P1/urea-SP3 protocol in different low numbers of dTHP1, THP1, and HT-29 cells. **(A)** Proteins identified per condition and cell type, with at least 2 unique peptides. **(B)** Peptides (unique and total) and peptide spectrum matches (PSMs) per condition. **(C)** Subcellular location distribution calculated as relative percentage (%) per total proteins. Protein locations were annotated according to UniProt database. Lists of proteins can be found in Supplementary Table 3 (dTHP1) and Supplementary Table 7 (THP1 and HT-29). In all panels, a reference (Ref) sample containing 20 μg of protein of each cell line was used as a control for maximum proteome coverage. *P*-values: * <0.05; ** ≤0.01; *ns*, not significant. For **(B)**: *, unique peptides; #, total peptides; $, validated PSMs. Significant differences were calculated using Kruskal-Wallis with Dunns' test for correcting for multiple comparisons.

For the evaluation of the P1-SP3 approach on small inputs, 2.5 and 10k cells from dTHP1, THP1 and HT-29 cell models were used. Additionally, a 20 μg-sample per cell line was also evaluated as a reference (Ref) for the complete proteome coverage. As observed in Supplementary Figure 2, very limited amount of protein was quantified and/or detected by silver staining (Supplementary Figure 3) in any of the cell lines at low cell numbers. However, the MS analysis of these samples (Figures 3A,B) reported 1,308 ± 141, 156 ± 45, and 266 ± 51 proteins (≥2 unique peptides) from 2.5k dTHP1, THP1 and HT-29 cells, respectively. The number of proteins identified increased 1.5–1.7x (1,962 ± 192, 265 ± 77, and 411 ± 49, respectively) (Supplementary Tables 3, 6) when no peptide/protein limit was set. In the same conditions, for 10k cells, almost 2x (for dTHP1) and 8x (for THP1 and HT-29) more proteins were detected (i.e., 3,478 ± 571, 2,019 ± 177, and 3,127 ± 164 proteins, respectively). Nevertheless, even when using a more stringent criterion for protein assignment (i.e., ≥2 unique peptides/protein), no significant differences were observed for 10 and 50k cells vs. Ref in all cases, and also for 2.5k vs. Ref for dTHP1 cells. Of note, proteome coverages from 50k cells and Ref sample were highly similar in terms of proteins identified. Correlation assessment among replicates (Supplementary Figure 5) reported a good reproducibility level (0.81–0.95, 0.84–0.94, and 0.86–0.94 ranges for dTHP1, THP1, and HT-29 cells, respectively) even at low cell numbers.

The distinctive proteins detected were assigned to their main subcellular location according to the UniProt database (Figure 3C). Overall, highly similar distributions were observed among the distinct dTHP1 cell numbers analyzed. Likewise, no significant differences were detected for THP1 and HT-29 cells, showing an almost identical layout for the 50k and Ref conditions. Despite the significant decrease in identified proteins when analyzing 2.5k cells, only minor differences related to nuclear (increased) and Golgi/mitochondria proteins (decreased) were observed compared to larger numbers of cells.

Furthermore, the functional annotation of small inputs (down to 10k) revealed no selective loss for any of the function groups, regardless of the cell type (Supplementary Figures 6B–D and Supplementary Table 6). Although THP1 and HT-29 showed a clear decrease in enriched pathways in 2.5k, this was not observed for dTHP1. Interestingly, the functional differences between cell lines were still observed from paucicellular samples, e.g., in HT-29, a relative increase in proteins involved in disorders of signal transduction, apoptosis and DNA repair was observed, whereas cell cycle functions were enriched in THP1, but not in dTHP1.

### Proteomic characterization of quantity-limited closely-related cells (PB monocyte populations and GBM MAC)

Monocytic populations (cMo, iMo and ncMo from six PB donors and MAC from five GBM patients), as well as T cells from both tissues as non-monocytic lineage models, were profiled at protein level on limited cell numbers (2.5 and 50k) by using the selected P1/urea-SP3 protocol.

**Figure 4:**
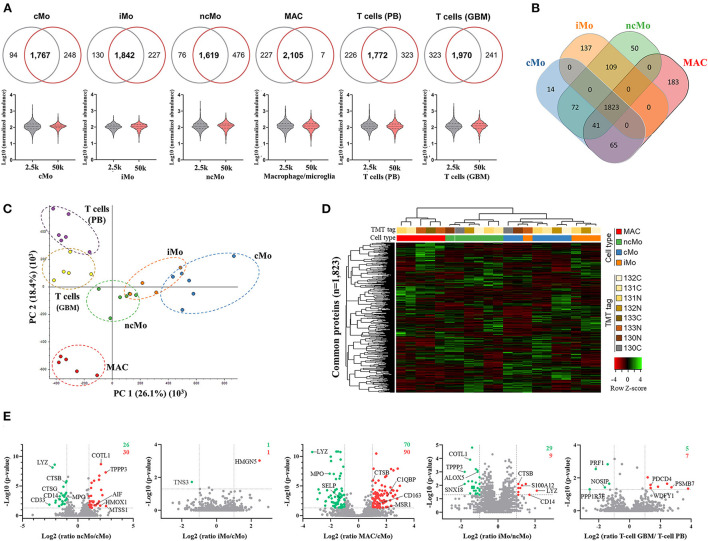
Quantitative proteomics analysis of the three major monocytic populations from peripheral blood of healthy donors and macrophages/microglia from glioblastoma patients. **(A)** Proteins systematically identified and quantified in all biological replicates in 2.5k (grey) and 50k (dark pink) cells. Venn diagrams (upper figure) display the overlapped proteins between the evaluated cell numbers, whereas the violin plots (lower figure) show the distribution of the quantitative data. **(B)** Venn diagrams of proteins identified and quantified in all donors, indicating common and distinctive proteins per population. **(C)** Principal component analysis (PCA) plot displaying the clustering of sample populations based on abundances of all proteins identified and quantified (*n* = 3,455) with at least two unique peptides and 1% FDR. **(D)** Hierarchical clustering analysis (using Pearson's correlation distance/complete linkage method) and heatmap comparing Z-scores from normalized expression values of proteins commonly expressed by monocytic populations (major populations and MAC). **(E)** Volcano plots showing log2 ratios of measured proteins in ncMo, iMo and MAC vs. cMo, iMo vs. ncMo, and T cells from GBM vs. T cells from PB. Green dots correspond to significantly (*p*-value < 0.05) downregulated proteins (ratio < 0.5), whereas red dots represent significantly upregulated proteins (ratio > 2). Numbers in the upper-right corner depict the total number of up- (red) and down-(green) regulated proteins per plot. *cMo*, classical monocytes; *iMo*, intermediate monocytes; *ncMo*, non-classical monocytes; *MAC*, macrophage/microglia; *GBM*, glioblastoma; *PB*, peripheral blood.

Up to ~4,000 proteins (with at least 1 unique peptide) were quantified across all samples (~3,200 proteins if ≥2 unique peptides/protein) (Table 1). However, to reliably characterize the monocytic populations, only proteins present in all donors and with at least 2 unique peptides were further considered (Supplementary Table 8). Data analysis of 2.5k vs. 50k cells (Figure 4A) uncovered a high overlap in identified proteins (average of 81.0% ± 5.8%), reaching 2,105/2,339 (90%) proteins in common for both cell numbers of GBM MAC. Furthermore, a similar protein abundance distribution was observed for both 2.5 and 50k cell conditions, with generally slightly more uniform data for 50k cells (Figure 4A, bottom graphs). When studying the protein distribution across monocytic subsets, 81.2% (1,823/2,246) proteins were commonly expressed in the major monocyte populations (Figure 4B), also showing a close cMo-iMo-ncMo connection in the PCA (Figure 4C). Moreover, although GBM MAC and monocytes shared a significant proteome (1,823/2,494; 73%) (Figure 4B), a clear separation between these cells was observed in the graph. Interestingly, cMo and GBM MAC shared the expression of 106 proteins, 41 of which were also expressed by ncMo (Figure 4B). Despite the abovementioned high overlap between the proteomes of monocytic subsets, protein expression levels differed among cell populations (Figure 4D) reflecting the dynamic regulation at the protein level within these cells. In particular, significant differences in protein expression (2-fold change) were observed for cMo vs. ncMo (56 proteins) and vs. GBM MAC (160 proteins) (Figure 4E). On the other hand, iMo and cMo appeared to be more similar, with only 2 proteins (TNSP3 and HMGN5) differentially expressed; whereas this number increased to 38 when iMo vs. ncMo were compared (Figure 4E).

**Table 1:** Number of proteins identified per condition (2.5 and 50k) and cell type (cMo, iMo, ncMo, MAC, PB T cells, GBM T cells).

**Cell population (cell number)**	**Number of identified proteins**			
	**Maximum**		**In all donors**	
	**At least 1 unique pp/protein**	**≥2 unique pp/protein**	**At least 1 unique pp/protein**	**≥2 unique pp/protein**
cMo (2.5k)	3,881	3,211	1,930	1,861
cMo (50k)	3,737	3,163	2,109	2,015
iMo (2.5k)	3,915	3,229	2,046	1,972
iMo (50k)	3,995	3,288	2,069	2,069
ncMo (2.5k)	3,903	3,224	1,758	1,695
ncMo (50k)	3,902	3,225	2,198	2,095
MAC (2.5k)	3,599	3,076	2,481	2,332
MAC (50k)	3,842	3,210	2,218	2,112
PB T cell (2.5k)	3,837	3,208	2,078	1,998
PB T cell (50k)	3,902	3,225	2,198	2,095
GBM T cell (2.5k)	3,600	3,075	2,430	2,293
GBM T cell (50k)	3,934	3,239	2,351	2,211

*Maximum* refers to proteins identified in any biological replicate, whereas *In all donors* refers to those proteins systematically detected in all biological replicates.

*cMo*, classical monocyte; *iMo*, intermediate monocyte; *ncMo*, non-classical monocyte; *MAC*, glioma macrophage/microglia; *PB*, peripheral blood; *GBM*, glioblastoma; *pp*, peptide.

**Figure 5:**
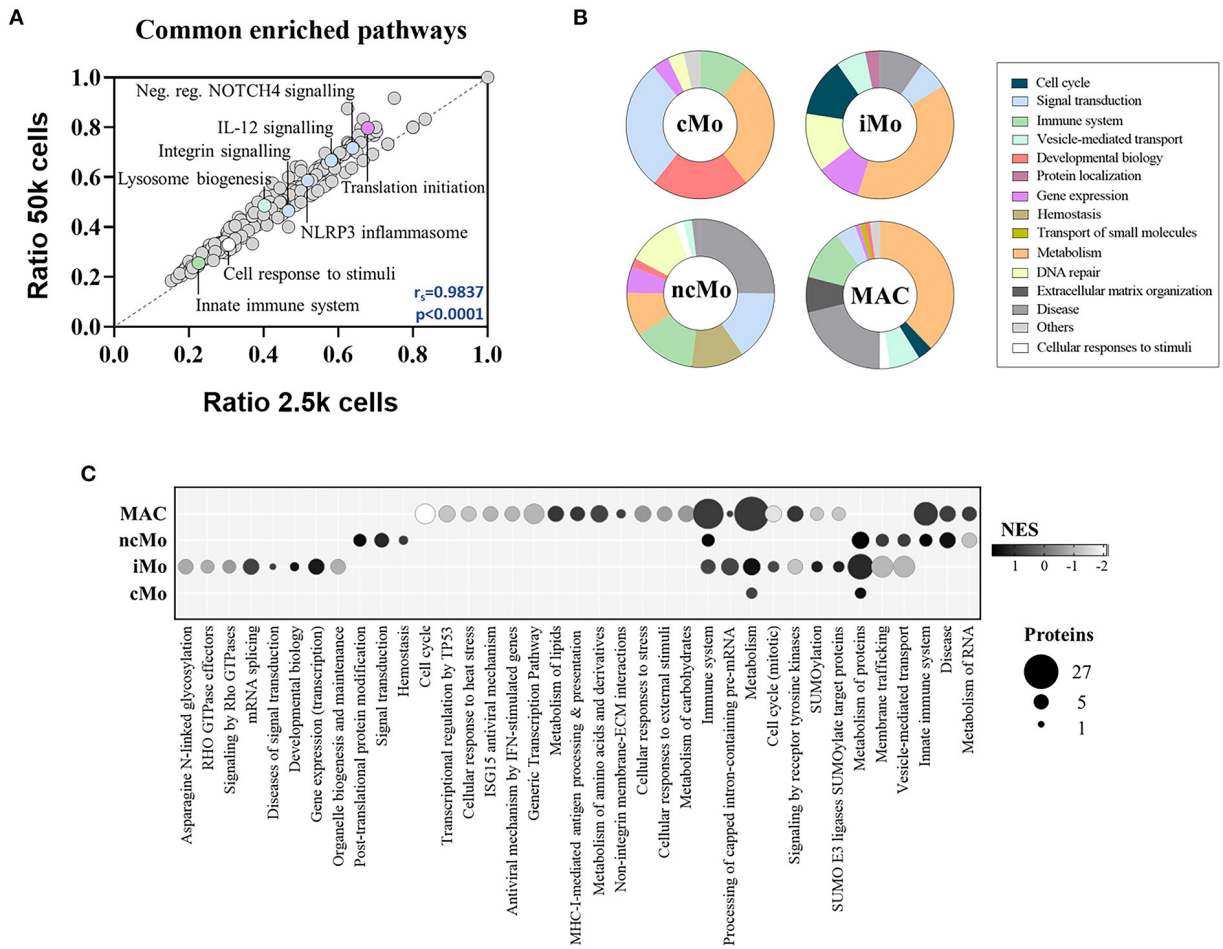
Functional enrichment analysis of the three major monocytic populations from peripheral blood of healthy donors and macrophages/microglia from glioblastoma patients. **(A)** Scatter plot showing the correlation between significantly enriched (*p*-value < 0.05) functional pathways in 50 and 2.5k cells from all studied populations, based on found entities/total defined ratios calculated from proteins in common for all populations. Spearman correlation coefficient (r_s_) and *p*-value (p) are indicated. **(B)** Main groups of functional pathways defined by unique proteins per population. **(C)** Selection of functional pathways determined by distinctive proteins per population. Bubble size is proportional to number of proteins, and bubble color indicates the normalized enrichment score (NES) calculated based on the protein expression data. *cMo*, classical monocytes; *iMo*, intermediate monocytes; *ncMo*, non-classical monocytes; *MAC*, macrophage/microglia.

The functional analysis of common proteins for monocytes and macrophages revealed the enrichment of regular cell functions, such as metabolism, protein translation and cell cycle, and other cell-specific activities such as signaling by interleukins and NOTCH4, membrane trafficking, antigen processing, MHC-I and MHC-II antigen presentations and Toll-like receptor cascades (Supplementary Table 9). Remarkably, a good correlation (r_s_ = 0.98) was found for all these pathways when comparing 2.5k- vs. 50k-cells-derived data (Figure 5A). On the other hand, the analysis of the distinctive proteins per population uncovered a diverse distribution of functions (Figure 5B). Metabolic pathways were represented within the four myeloid subsets, with higher coverage in iMo and GBM MAC. Conversely, signal transduction functions were further depicted in cMo and ncMo. Figure 5C shows a deeper analysis of the pathways, number of distinctive proteins and enrichment scores per population.

As for T cells (data not shown), more than three-quarters of total T cell proteins (1,863/2,441; 76%) were in common regardless of the tissue origin (i.e., PB or GBM), grouping close to each other in the PCA (Figure 4C) and depicting similar protein expression levels. However, differences were also observed as 231 and 347 proteins were expressed by T cells from PB and GBM, respectively, but not by their corresponding counterpart. A functional enrichment analysis of these proteins revealed that T cells infiltrating GBM are involved in multiple signal transduction pathways (e.g., PI3K/AKT, VEGF, PDGF, FGFR1, MAPK), whereas those from healthy PB are engaged in more steady-state functions such as mRNA splicing, showing the influence of the (tumoral) microenvironment in their cell proteomes.

### Comparison of the selected P1/urea-SP3 method with other studies

To demonstrate the relevance of our data, we compared the obtained results with those from other studies. For instance, Ravenhill et al. (37) recently reported the surface proteome of cMo by biotinylating the membrane of 10^7^ cells to further perform TMT-based MS proteomics. A total of 373 proteins were identified, with 22 contributing to almost 70% of the surface proteome. In our study, using 4,000x fewer cells and without any enrichment step, 82% (18/22) of these proteins were identified (100% when using 50k cells). Likewise, Soday et al. (38) defined 593 plasma-membrane annotated proteins in cMo by following an analogous biotinylating-based strategy, of which 347 (contributing to 88% of cMo surface proteome) were detected from 2,500 cells in our report. Interestingly, Zeng et al. (39) claimed to have profiled the monocyte proteome knowledge base; however, monocyte samples only consisted of cMo contaminated with basophils and dendritic cells, and relaxed criteria were used for protein selection (i.e., 4% FDR, 1 peptide/protein). Their results reported 2,237 proteins, but only 1,461 (39%, 1,461/3,737) matched our data from FACS-purified cMo (purity>96.4%; 2 pp/protein, 1% FDR, proteins present in all donors) highlighting the importance of high-quality samples and strict selection criteria to define cell reference maps. As for studies on minute amounts of macrophages, our study outperformed the results of Sielaff et al. (26) who evaluated and modified the SP3 methodology in bone marrow-derived macrophages from mouse. Whereas, they identified 3,152 proteins from 25,000 cells, our study reported a similar number (i.e., 3,076 proteins) from 10x fewer macrophages. Considering the SP3 sample clean-up strategy and the data analysis parameters were highly similar, the usage of a different lysis buffer and mass spectrometer certainly influenced the performance. Muller and colleagues (40) used a protocol similar to our P3/SDS-SP3 coupled to a liquid handling robot (autoSP3) reporting 500–1,000 proteins from 100 to 1,000 HeLa cells, supporting once more the good functioning of the bead-based system. When referring to studies oriented toward single-cell analysis, technologies such as SCoPE2 (15) allowed for quantification of 3,042 proteins from 1,490 monocytes and macrophages, which outperformed our method; however, 10 days of instrument time were required for such analysis, whereas in our case only 12 h were needed. Similarly, Budnik et al. (14) used the SCoPE-MS system to quantify 767 proteins from single cells; however, 200 cells were added as carriers for each cell of interest to be measured (introducing potential noise) and thousands of single cells had to be measured to obtain reliable results (with a cost of $15–30 per cell). Remarkably, another study based on SCoPE-MS (15) reported comparable results to our data, as 2,700 proteins were identified from only 1,018 cells (in our study 3,881 proteins were detected from 2,500 cells). Similar results were also obtained with the SOPs-MS (surfactant-assisted one-pot sample processing at the standard volume coupled with MS) method (41).

### Proteomics validation by high-end spectral flow cytometry

**Figure 6:**
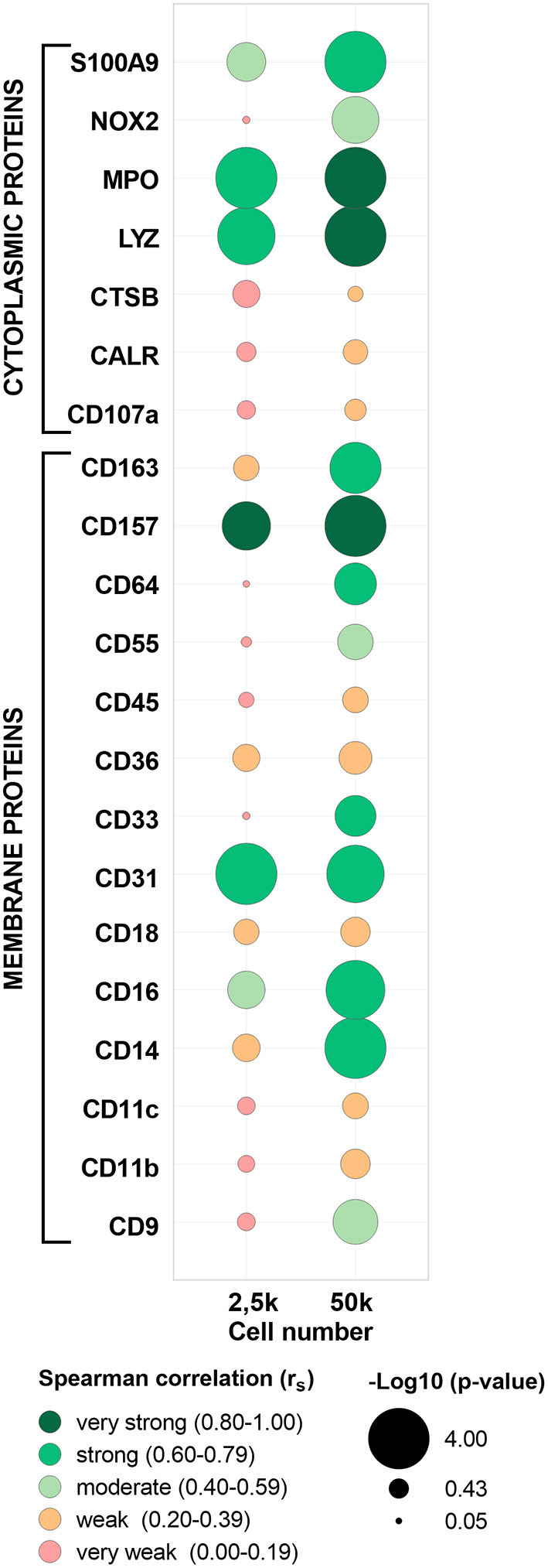
Correlation between abundance ratios (vs. classical monocytes, cMo) of selected proteins from paired samples in flow cytometry (FCM) and mass spectrometry (MS). Each bubble includes data for each protein and cell number (2.5 or 50k) from cMo, non-classical monocytes, intermediate monocytes, glioblastoma macrophages/microglia and T cells from peripheral blood. Bubble colors indicate the Spearman correlation value and bubble size is proportional to -log10 (*p*-value) as indicated below the panel. CD68 and CD282 proteins were not included as no quantification values were calculated by MS. *MPO*, myeloperoxidase; *LYZ*, lysozyme C; *CTSB*, cathepsin B; *CALR*, calreticulin.

To further validate the MS/MS data, a highly sensitive antibody-based method (i.e., FC) was applied in paired samples of the five evaluated populations (cMo, iMo, ncMo, MAC from GBM, T cells from PB and GBM) to determine the expression of 23 markers (15 membrane and 8 cytoplasmic proteins, Supplementary Figure 8 and Supplementary Table 1). First, FC data was compared *vs*. 50k-derived MS data (Supplementary Table 10) uncovering a good concordance (>75%) in protein detection (presence/absence) for tested replicates across populations between both techniques for 16/23 (70%) markers. Seven markers (CD9, CD11c, CD18, CD45, CALR, MPO, S100A9) even showed a 100% concordance between FC and MS. Additionally, expression ratios (vs. cMo) calculated either using the mean fluorescence intensity for FC and the normalized abundance values for TMT-based MS (Figure 6) reported a moderate (r_S_: 0.49) average correlation for all evaluated markers, with 11/23 (48%) proteins depicting strong (r_S_: 0.60–0.79) to very strong (r_S_: 0.80–1.00) correlations. Moreover, the two -omics techniques performed quite similarly for cytoplasmic and membrane proteins (average r_S_ 0.47 ± 0.27 vs. 0.50 ± 0.25, respectively); however, some membrane markers (CD33, CD55, CD64, CD157) showed poor results as they were not consistently identified by MS and two proteins (CD68 and CD282) did not pass the signal-to-noise threshold of the mass spectrometer equipment. Remarkably, the presence/absence concordance for FC vs. MS was maintained when evaluating 2.5k cells, also including seven markers with 100% agreement between both -omics. Conversely, overall correlation values were weak (average r_S_: 0.30), although 4 markers (CD31, CD157, LYZ, MPO) presented strong to very strong correlations.

### Confirmation of broad applicability of P1/urea-SP3 method in other cell types

To prove the broad applicability of the method, we applied the P1/urea-SP3 procedure for the analysis of 38 different cell types (*n* = 194 samples) with different tissue origins (PB, bone marrow, skin, colon, peritoneal dialysate), phenotypes (normal, tumoral), lineages (myeloid, lymphoid, epithelial), pre-processing (sorting, no sorting), experimental (*in vitro, ex vivo*) and stimulation (viral, bacterial, unstimulated) conditions (Supplementary Table 11 and Supplementary materials and methods). First, the P1/urea lysis protocol was tested showing a good performance across all cell types, regardless of the sample size and even at very low cell numbers (down to 263 cells). Protein amounts per cell were donor- and cell type-dependent (e.g., in bone marrow, monoblasts expressed more protein amount vs. mature cMo). Twenty-four of these populations (*n* = 112 samples) were further processed with the SP3 approach for MS analysis (using 4 μg of protein per sample) reporting excellent results (3,700 ± 359 proteins) and confirming the broad applicability of the selected method.

## Discussion

In the era of single-cell studies, new proteomics approaches have been developed allowing e.g., profiling of the phenotypic heterogeneity within the same cell population. However, these single-cell-oriented strategies require highly (often home-built) costly special devices and complex non-standardized protocols, hampering their routine application in translational research and clinical settings, which require high-throughput, easily applicable and reproducible approaches. Profiling of individual cells has obvious benefits, but it will be advantageous to have a more affordable method, that is standardized, easy-to-use and widely applicable in non-specialized proteomics environments and that can reliably characterize paucicellular (closely-related) samples. With this goal in mind, we assessed 10 combinations of five lysis buffers (P1–P5) with two sample clean-up techniques [SP3 and C18 (23, 24, 30)] via a stepwise approach structured in three phases. In the technical assessment stage, cell lines for monocytes, macrophages and colon cancer cells were processed to define the best procedure in a quantity-limited setting. In the application stage, the three major monocyte populations from PB and MAC from GBM (and T cells from both tissues) were used as models to in-depth evaluate the usefulness of the method for determining differences between closely maturational-related cells. In the validation phase, the feasibility of the selected method was proven in 38 different cell types, and its performance was tested by high-end spectral FC.

Cell type features determine lysis efficiency and lysis reagents might affect MS analysis (42). Therefore, the choice of the lysis method is crucial to achieve optimal outcomes and must be evaluated case-by-case. In this study, five lysis buffers were selected based on their known performance and mechanism for extraction of proteins. Overall, the methods employed included urea as a denaturant reagent [P1 (26), P2 (27)], which disrupts hydrogen bonding between amino acids weakening the protein hydrophobicity; SDS as a detergent [P3 (23)] that establishes hydrophobic interactions with proteins and hydrophilic links with water; TFE as organic cosolvent [P4 (28)] which destroys tertiary structures by weakening hydrophobic interactions and disrupting water networks; and a hypotonic solution (P5), which causes cells to swell as a result of osmotic diffusion, leading it to burst, and therefore releasing the protein content. Assessment of the abovementioned approaches revealed different performances depending on the cell type and number. Overall, P2–P4 cell lysis generally resulted in better protein extractions when compared to P1 (up to 3 folds), suggesting that the usage of detergents and cosolvents allows for better protein solubilizations and recovery as demonstrated by Ashraf Kharaz et al. (43) and Proc et al. (44), respectively. Furthermore, even though both P1 and P2 lysis solutions contained urea as main component, the amount of extracted protein differed 2x for THP1 cells, corroborating that the selection of the buffer (HEPES vs. TEAB, for P1 and P2, respectively) and the addition of protease/phosphatase inhibitors (45, 46) are also relevant for the lysis. Remarkably, P5/hypotonic performed the worst on 50k cells but could extract quantifiable protein amounts in lower cell numbers (together with P4/TFE) suggesting that more complex lysis buffers might either be ineffective in low ranges due to sample losses and/or could affect protein quantification (28, 47). Silver staining and MS analysis confirmed the presence of proteins in non-quantifiable samples, supporting the latter hypothesis.

As for clean-up methods, SP3 outperformed C18 in all studied cases, regardless of the lysis buffer used, depicting the advantages of this single-vessel paramagnetic bead-based system where the hydrophilic interaction between carboxylate-coated beads and proteins (via ethanol-driven solvation capture on the bead surface) permits removal of contaminants without compromising the peptide purification in limited samples by elution in aqueous conditions (24). Surprisingly, despite the quantification differences observed for P1 vs. P2–P4 methods, similar numbers of identified proteins were detected. This was probably due to impurities from buffers P2–P4 still present in the final sample to be measured and/or specific lysis buffer-dependent proteins that were highly represented in those mixtures. Also, notwithstanding the good performance of P2 in dTHP1 cells, a poor inter-replicate correlation (r^2^ = 0.44) after MS analysis was observed presumably due to buffer variation over time caused by the volatile nature of TEAB (48). For P5/hypotonic method, no significant improvement was observed, further highlighting the need to include a strong molecule to enhance protein extraction. Of note, the poor performance of P3/SDS-C18 in low cell numbers was most likely due to filter clogging (49) caused by the high SDS percentage and the fact that this detergent can elute with peptides affecting the MS analysis. The addition of extra clean-up steps as those of the FASP procedure (22) to eliminate the SDS might result in better outcomes; however, this approach is time-consuming and requires sample transfer steps [even in its micro version developed by (50)], which can potentially affect the recovery of the peptides.

Even though each P1–P4 SP3 combination identified a similar number of proteins in dTHP1 cells, only 40% of the total proteome (5,975 proteins) was detected in common to these approaches, reflecting the different protein selectivity per method. Therefore, to reliably select the best approach for paucicellular samples, not only raw protein identification numbers must be considered, but also protein types based on their subcellular location and function. As for the former, it was shown that the P1/urea method better reflected the overall actual spatial distribution of the proteins, not enriching for any particular compartment and with a clear better representation of membrane and nuclear proteins as compared with other protocols. As for the protein functions, the monocytic model cells used here are known for their phagocytic and antigen presentation activities (51), being the detection of related proteins key to characterize them. In this sense, the P1/urea approach allowed for the identification of relevant membrane macrophage proteins, such as chemokine receptor 1 (CCR1) (52) and HLA-E molecule (53) whose expressions have been described to be increased in the monocyte-macrophage differentiation, and IL-6 receptor (54), restricted to macrophages vs. monocytes (not detected in THP1 cells). Thus, considering the high identification efficiency for 50k macrophage-like cells (similar proteome coverage as 20 μg-reference sample), the correspondence with actual subcellular location distribution, the great reproducibility, ease of use, the time needed and cost per sample, the combination P1/urea-SP3 was selected as the best approach.

Aiming at testing the performance of this method at even smaller inputs, 2.5 and 10k cell samples were processed. Although an expected decrease in identified proteins was observed when lowering the cell numbers, up to 1,308 and 2,281 proteins (1,962 and 3,478 when >1 unique peptide) were detected on average for 2.5 and 10k cells, respectively. Also, a high inter-replicate correlation was observed in these samples. The functional cell characterization and protein subcellular location distribution remained unchanged, indicating the P1/urea-SP3 method does not introduce bias in paucicellular samples. Interestingly, differences between cell types could still be determined in lower cell numbers. Thus, dTHP1 showed more enriched pathways related to immune system-related functions, transport and trafficking compared to THP1, in line with the more mature maturational stage of dTHP1 (i.e., macrophages) and the immune-activated status of mature monocytes (55–58). Conversely, THP1 cells (i.e., monoblasts) were enriched in proteins for cell cycle division, typically associated with more immature cells (58).

Despite major monocytic populations and GBM macrophages have been extensively studied by different approaches (51, 59–64), including MS-based proteomics strategies (37, 39, 65–69), none of these analyses has been performed in a quantity-restricted setting. Therefore, a 2.5k-cell number lower limit was set for investigating the cMo, iMo, and ncMo subsets from PB and MAC from GBM samples, together with T cells from both tissues as an internal control. Interestingly, the P1/urea-SP3 methodology performed better in the *ex vivo* samples compared to the cell line assessment, showing an 80% average overlap between 2.5 and 50k conditions for all populations. Hence, biological analysis was based on 50k cell-derived data.

The monocyte-macrophage protein signature was defined by 1,823 proteins (detected with ≥2 unique proteins, in all donors). Their expression modulated between populations clearly distinguishing three groups: *(i)* GBM MAC, distinct from the other monocytes, *(ii)* ncMo, and *(iii)* cMo+iMo. Of note, ncMo appeared as the closest cell subset to GBM MAC suggesting a cMo-iMo-ncMo-macrophage maturation trajectory. The functional analysis of the commonly expressed proteins revealed the involvement of monocytic cells in 479 enriched pathways (*p*-value < 0.05), including not only standard cell functions (e.g., cell cycle, translation initiation) but also cell-specific pathways. Among them, the *NLRP3 inflammasome*, involved in the release of pro-inflammatory cytokines IL1β and IL18 and whose expression in immune cells is limited to the myeloid compartment (70, 71); and the *IL-12 signaling*, triggered by monocytes/macrophages in response to pathogens via Toll-like receptors inducing the adaptive immune response (72). As for the *Integrin signaling* enriched cascade, it is well-known that monocytes express these proteins favoring cell adhesion, migration through the endothelium, phagocytosis and proliferation (73). However, their expression levels differ between subsets as it has also been shown in this study for CD11a, CD11b, CD11c, and CD18, with lower levels at GBM MAC than monocytes. On the contrary, CD11d, whose mRNA levels have been reported in monocytes by Villani et al. (51), has not been detected in our study at the protein level. These observations were confirmed by previous proteomics reports on monocytes employing higher cell numbers (10-66x more cells) (74). Also, *Lysosome vesicle biogenesis* and other related pathways (*ER to Golgi transport, ER-Phagosome pathway, lysosome transport*) were enriched in monocytes/MAC depicting the role of the secretory cascade and the enzyme production for further protein digestion of pathogens/damaged cells in phagolysosomes.

Despite the high proteome overlap (73%), each monocytic cell population exclusively expressed several distinctive proteins. For cMo, 14 proteins were found (TAF10, CLC, UGDH, PAK1, UBFD1, CAVIN1, TBCD, P3H1, CHKB, ZMYND8, ACTR5, RANBP3, HMBS, VPS26C), mainly involved in signal transduction and metabolism processes. Of note, CLC (a.k.a. galectin-10), which regulates immune responses via surface glycan recognition and is essential for the anergy and suppressive function of CD25^+^ regulatory T cells, has been reported to be exclusively expressed by eosinophils and basophils in other studies (75–77). However, we have demonstrated its presence in monocytes. Importantly, the detection of Ser/Thr kinase PAK1 that relates to cytoskeleton dynamics, cell adhesion, migration, proliferation and vesicle-mediated transport, together with the overexpression of cell motility-related molecules (PDLIM1, VCAN, PXN, ITGAM, VCL, CTTN, NEXN, ILK, MAPRE2), supported the canonical role of phagocytosis assigned to cMo. Also, microbicidal enzymes such as MPO, CTSG and LYZ were remarkably overexpressed compared to ncMo and GBM MAC. Likewise, expression of pro-coagulation- (MMRN1, THBS1, ITGB2/ITGA2B, SELP, GP6, F11R, GP5, FGA/B, HPSE, VWF, VASP) and pro-inflammatory-related (S100A8, S100A9) proteins was enhanced in these cells (vs. ncMo and/or macrophages). C-lectin receptors, such as ficolin-1 which recognizes pathogen-associated molecular patterns (PAMPs) and may also induce the secretion of IL-8, and the CD36 scavenger receptor, were found overexpressed vs. GBM MAC. Conversely, CTSB expression was higher in the latter subset. Interestingly, cMo also showed patrolling capabilities by the expression of transendothelial migration-related F11R and PECAM1 proteins (at higher and similar levels vs. ncMo, respectively).

On the other hand, iMo showed upregulation of metabolic, gene expression, cell cycle, and DNA repair processes, depicting the metabolic active behavior of these cells. Moreover, only two proteins showed significantly different expression levels vs. cMo, while 38 markers were distinctly expressed between iMo and ncMo displaying a more similar phenotype to the former subset. Considering previous suggestions that iMo might not comprise a fully independent cell subset but a continuation of the cMo to ncMo differentiation (78, 79), it appears logical that these cells present heterogeneous profiles. That is the case for inflammation processes since overexpression (S100A12, CD36) and underexpression (S100A6, ALOX5) of several pro-inflammatory markers was observed. Unlike ncMo, iMo overexpress CTSB, LYZ, CD14 and PXN. Also, while iMo have been classically described to have increased T-cell stimulation function, HOMER3 protein, a negative regulator of T-cell activation, was highly present in this subset, whereas SPN, AIF1 and ADA (related to positive regulation of T-cell coactivation and/or proliferation) were downregulated.

The ncMo subset displayed the distinctive expression of 50 proteins involved in homeostasis (OTULIN), signal transduction (ERBIN, NMI, CFP), DNA repair (BRD2, SDE2), metabolism of proteins (MCCC2, CUL3, PPIL2, GZMM) and RNA (RAB44, ISY1), and vesicle-mediated transport (STX4, CHMP5, GAS2L1, MAP1A, RUFY3). The detection of SKAP1, which regulates the T cell-APC conjugation, and RPS6KA5, usually expressed in macrophages to limit the production of pro-inflammatory cytokines, supported the abovementioned closer profile of ncMo to macrophages. Also, overexpression vs. cMo of proteins related to apoptosis (ANP32A, CPPED1, ASAH1), cell migration (MARCKSL1, ALOX5, MTSS1, S100A6, TPPP3), anti-viral responses (ISG15, GBP1) and T-cell stimulation (LCP1, ADA, AIF1) was previously shown (40, 80). Special attention requires AIF1, a calcium-binding protein expressed in macrophages, microglia and DCs, and involved in phagocytosis, inflammation, antigen presentation and T-cell polarization (81). AIF1 is important for monocyte conversion into pro-inflammatory macrophages; however, little is known about its expression within the monocyte subsets. Previous analyses (82) have shown higher RNA levels in iMo>ncMo>cMo. Although the lowest protein levels of AIF1 were also detected in cMo, an opposite pattern was found for iMo and ncMo, with the latter showing an identical pattern to GBM MAC. The same profile was observed for the pro-inflammatory immunoregulatory amidase NAAA. Expression of S100A6, a calcium sensor/modulator, uncovered controversial outcomes since previous transcriptomics and proteomics studies (74) indicated higher levels in cMo vs. ncMo, whereas our data clearly showed the opposite result, suggesting that their transcript and protein levels do not correlate.

In GBM, there is a mix of mononuclear antigen-presenting phagocytes, sharing functional profiles and large sets of proteins. Also, marker co-expression in the same cells has been previously observed, supporting the idea that they might comprise a mixed population (83). In this study, three out of five GBM MAC samples consisted of >96% microglia cells. The GBM MAC proteome, profiled for the first time in this study, reported the clustering of these samples based on the abovementioned cell composition, being the highly pure microglia samples closer to ncMo. Overall, GBM MAC were significantly enriched in functions as *vesicle-mediated transport, scavenging by class A/H receptors, IFN and PD-1 signaling*, and *integrin cell surface interactions*. A deeper analysis revealed a mixed proteomics M1/M2 profile [previously observed at RNA level (84, 85)], with a higher representation of proteins associated with M2 macrophages showing a more pro-tumoral and anti-inflammatory phenotype. Although some M1 (CCL5, CD64, HLA-II molecules) and anti-tumoral (ADAM10, ADAM17, SCIN) markers were detected, the majority of GBM MAC expressed M2-linked proteins as CD14, CD36, CD163, CD204, CD206, TGFβ and HLA-II molecules (HLA-DBQ1, -DPA1, -DPB1, -DRB1, DRB3, -DRB5, CD74), together with other anti-inflammatory (FCER1G, TYROBP, SIGLEC9/10) and angiogenesis-stimulating/pro-tumoral surviving (SPP1) markers. Other M2-related functions (83, 86) were annotated, e.g., phagocytosis/digestion (CD11b, CPVL, RAB32, CTSB, CPQ), degradation (GAA, GLA, GUSB, BLAT1, HSPA5, VPS4A, PPT1) and homeostasis restoration (AQP4, FTH1, FTL).

Finally, we validated 15 membrane and 8 cytoplasmic proteins by FC reporting an overall optimal identification performance of MS on paucicellular samples, except for a few membrane markers (i.e., CD33, CD55, CD64, CD157) that were not systematically detected in all donors. As expected, several membrane-bound proteins were more difficult to extract and, therefore, to identify by MS (87); however, by using membrane-targeted lysis methods, this correlation might significantly improve. As for the expression quantification, even though an increased sensitivity was observed for FC (due to better performance of antibodies vs. mass tags), strong-to-very strong correlations between both -omics were defined for almost half of the tested proteins.

Nevertheless, the advantages of MS-based methods for comprehensive proteome characterization are undeniable. In this particular study, we have demonstrated the reproducibility, high performance, cost and time effectiveness, ease of use and broad applicability of combining a urea-based lysis buffer with the SP3 sample clean-up method for the reliable profiling of paucicellular closely-related populations. Our results demonstrate complete proteome landscapes, even for 2.5k cells, allowing the evaluation of protein expression modulation between monocytes and macrophages and definition of functional features per cell type. Additionally, GBM-purified MAC and T cells have been characterized at the protein level for the first time, and a demonstration of the broad applicability of the method was conducted in 38 different cell types across 194 donor samples. Even though other MS-based approaches might enable working at a single-cell level (14, 15), these methods are still highly variable, time-consuming (88), expensive, require complex devices and/or do not offer complete proteome coverages, hampering their application in translational research and clinical settings (46) (Supplementary Table 4). By contrast, the here proposed method offers the possibility of further developing the clinical MS field for studying e.g., the immune system, since limitations in sample material and access to the required MS equipment are no longer a barrier.

## Data availability statement

The datasets presented in this study can be found in online repositories. The names of the repository/repositories and accession number(s) can be found at: http://www.ebi.ac.uk/pride/archive/, PXD018872, PXD026604.

## Ethics statement

The studies involving human participants were reviewed and approved by LUMC Volunteer Donor Service, B18.031, project request L18.001 (for peripheral blood samples); Medical Ethical Committees of Erasmus Medical Center Rotterdam, 2013-139 (for glioblastoma samples); LUMC 2018.IMM.BM (for bone marrow samples); NVT0532.01, Sanquin Blood Bank (for buffy coats); B18.009 (for skin samples); W2017.031 (for peritoneal dialysate samples); NL66875.056.18 (for colon samples). The patients/participants provided their written informed consent to participate in this study.

## Author contributions

PD, CT, and JJMvD designed the study. KvdP, SK, IK, FaM, ALdJ, BAEN, IdL, AL, CT, and PD contributed to the collection, processing, and/or analysis of all samples. ES,WBLvdB, LBV, RKB, and MLML provided the glioblastoma samples. AHdR, GMCJ, RTNT, and PAvV performed the mass spectrometry measurements. AO contributed to the flow cytometry validation. KvdP and PD wrote the manuscript. All authors contributed to the manuscript revision and approved the submitted version.

## Funding

The presented work was funded by the European Research Council under the European Union's Horizon 2020 Research and Innovation Programme with an ERC Advanced Grant (ERC-2015-AdG 695655, TiMaScan).

## Conflict of interest

Authors JD and AO are chairmen of the EuroFlow scientific foundation, which receives royalties from licensed patents, which are collectively owned by the participants of the EuroFlow foundation, to be exclusively used for the continuation of the EuroFlow collaboration and sustainability of the EuroFlow consortium, originally supported by the European Commission (EU-STREP Project LSHB-CT-2006-018708). Authors JD and AO report an Educational Services Agreement from BD Biosciences (San José, CA) and a Scientific Advisor Agreement with Cytognos; all related fees and honoraria are for the involved university departments at Leiden University Medical Center and University of Salamanca. The remaining authors declare that the research was conducted in the absence of any commercial or financial relationships that could be construed as a potential conflict of interest.

## Publisher's note

All claims expressed in this article are solely those of the authors and do not necessarily represent those of their affiliated organizations, or those of the publisher, the editors and the reviewers. Any product that may be evaluated in this article, or claim that may be made by its manufacturer, is not guaranteed or endorsed by the publisher.

## Abbreviations

ACN: acetonitrile
cMo: classical monocytes
dTHP1: THP1 cells differentiated with PMA
FASP: filter-aided sample preparation
FC: flow cytometry
FDR: false discovery rate
GBM: glioblastoma
iMo: intermediate monocytes
MAC: macrophages/microglia isolated from GBM samples
MS: mass spectrometry
ncMo: non-classical monocytes
NSAF: normalized spectral abundance factor
PB: peripheral blood
PBMCs: peripheral blood mononuclear cells
PCA: principal component analysis
PMA: phorbol 12-myristate 13-acetate
PSM: peptide spectrum match
RT: room temperature
SP3, single-pot: solid phase-enhanced sample preparation
TEAB: triethylammonium bicarbonate
TFE: trifluorethanol
TMT: tandem mass tag.
