# Supplementary material for: Quantitative proteomics of small numbers of closely-related cells: Selection of the optimal method for a clinical setting
Source: Front Med (Lausanne). 2022 Sep 27;9:997305. doi: 10.3389/fmed.2022.997305 (PMC9553008; doi:10.3389/fmed.2022.997305)
Supplement: Supplementary file 1 [file Data_Sheet_1.zip › 997305_Supplementary Material/Supplementary Figures.docx]

**Supplementary Material**

**Quantitative proteomics of small numbers of closely-related cells: Selection of the optimal method for a clinical setting**

Kyra van der Pan^1^, Sara Kassem^1^, Indu Khatri^1,2^, Arnoud H de Ru^3^, George MC Janssen^3^, Rayman TN Tjokrodirijo^3^, Fadi al Makindji^1^, Eftychia Stavrakaki^4^, Anniek L de Jager^1^, Brigitta AE Naber^1^, Inge F de Laat^1^, Alesha Louis^1^, Wouter BL van den Bossche^4^, Lisette B Vogelezang^4^, Rutger K Balvers^4^, Martine LM Lamfers^4^, Peter A van Veelen^3^, Alberto Orfao^5^, Jacques JM van Dongen^1,5^, Cristina Teodosio^1,5†^, Paula Díez^1,5†^

^1^ Department of Immunology, Leiden University Medical Center (LUMC), Leiden, The Netherlands

^2^ Leiden Computational Biology Center, LUMC, Leiden, The Netherlands

^3^ Center for Proteomics and Metabolomics, LUMC, Leiden, The Netherlands

^4^ Department of Neurosurgery, Erasmus MC, Rotterdam, The Netherlands

^5^ Translational and Clinical Research Program, Cancer Research Center (IBMCC; University of Salamanca - CSIC); Cytometry Service, NUCLEUS; Department of Medicine, University of Salamanca and Institute of Biomedical Research of Salamanca (IBSAL), Spain

† These authors share last authorship

**Correspondence:** Prof. J.J.M van Dongen, MD, PhD

Leiden University Medical Center (LUMC)

J.J.M.van_Dongen@lumc.nl

**SUPPLEMENTARY FIGURES
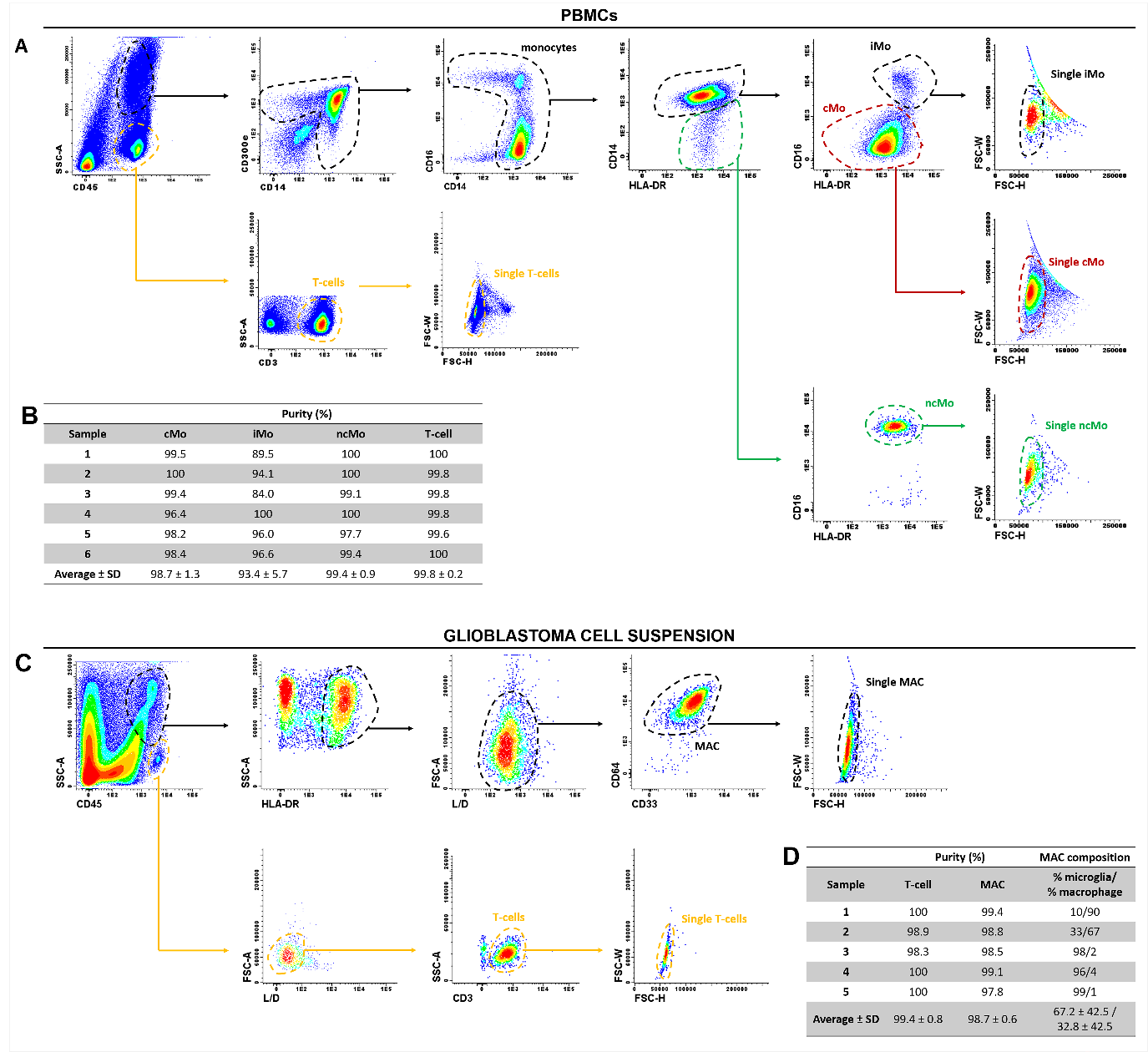
**

**Supplementary Figure S1.** Gating strategy of peripheral blood mononuclear cells (PBMCs) and glioblastoma cell suspensions for isolation of monocyte subsets, macrophages/microglia (MAC) and T cells. A) Gating strategy used for isolation of cMo (red), iMo (black) and ncMo (green) as well as T cells (yellow) from PBMCs. B) Sort purities of cMo, iMo, ncMo and T-cell populations obtained from 6 PBMC donors, including the average purity (mean ± SD). C) Gating strategy for isolation of MAC (black) and T cells (yellow) from glioblastoma cell suspensions. D) Sort purities of MAC and T cell populations obtained from 5 glioblastoma donors, including the average purity (mean ± SD). The exact composition of the MAC compartment is indicated in the right column. Definition of macrophages vs microglia was performed based on CD45low (microglia) vs CD45high (macrophage) within HLA-DR+, CD33+ and CD64+ gate. cMo, classical monocytes; iMo, intermediate monocytes; ncMo, non-classical monocytes; MAC, macrophages/microglia; SD, standard deviation.

**
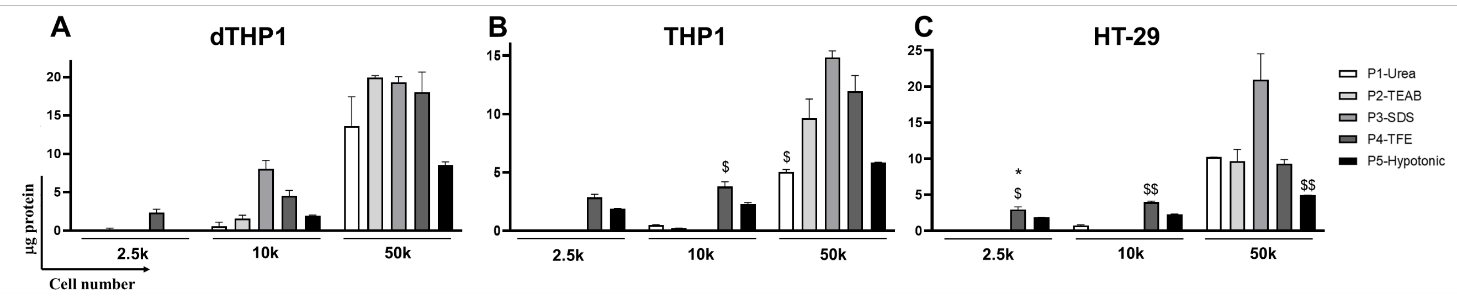
**

**Supplementary Figure S2.** Protein extraction efficiency of cell lysis protocols in different cell numbers and cell types. Quantified extracted protein (in µg) by urea- (P1), TEAB- (P2), SDS- (P3), TFE- (P4), and hypotonic- (P5) based protocols in 2.5k, 10k and 50k cells of A) phorbol 12-myristate 13-acetate (PMA)-differentiated THP1 (dTHP1), B) THP1, and C) HT-29 cell lines. Median with range is shown per bar. Statistically significant differences were evaluated using the Kruskal-Wallis test with the Dunn’s test to correct for multiple comparisons vs *P1; $P3. P-values: *<0.05; **<0.01.

**
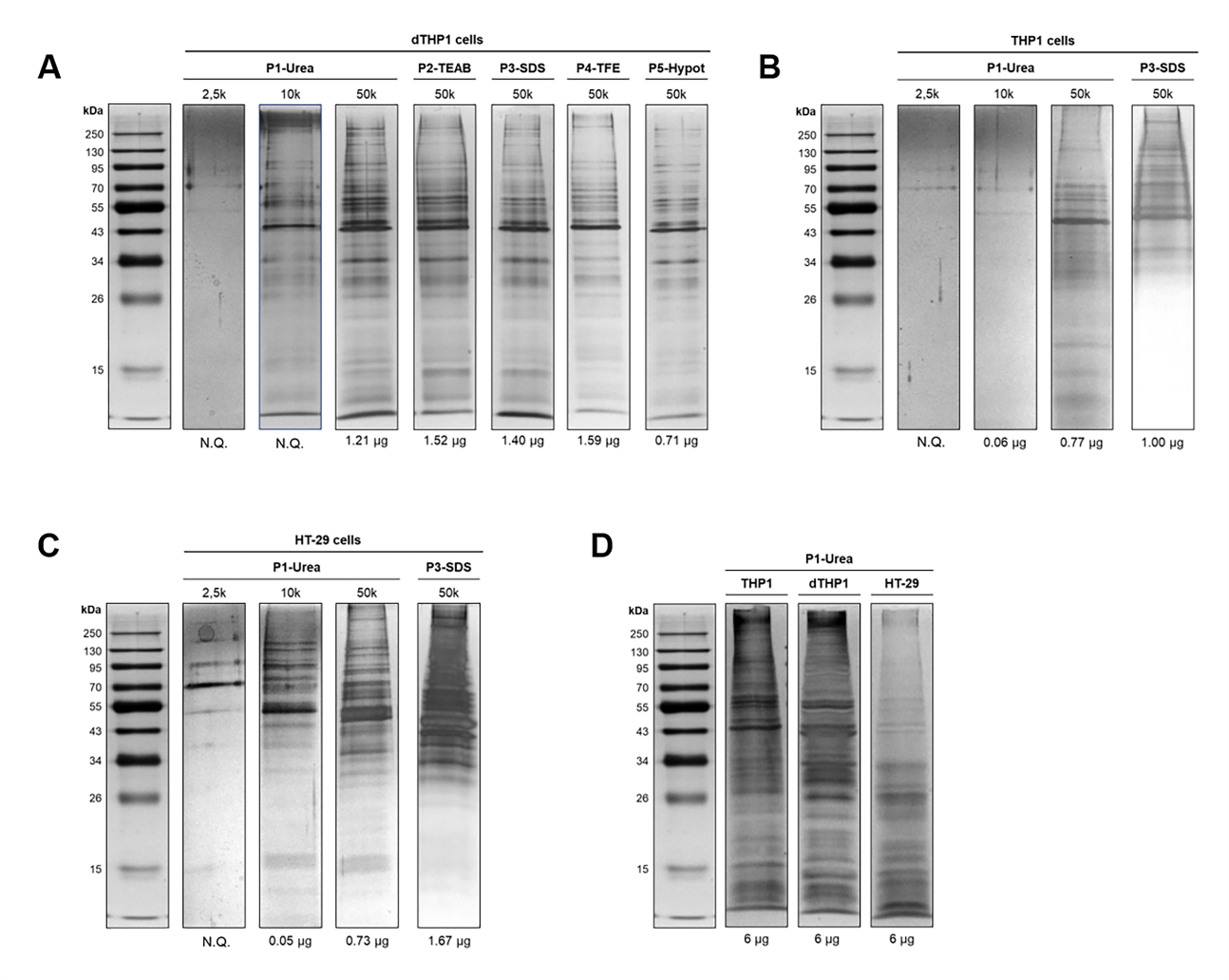
**

**Supplementary Figure S3.** Silver-stained SDS-PAGE gels of different A) dTHP1, B) THP1 and C) HT-29 cell numbers lysed with protocols P1-P5. Same sample volume (6 μL = 2 μL x 3 replicates) was loaded per condition. In Panel D) 6 μg of each cell line was loaded as reference. Numbers below each gel lane indicate the loaded protein amount. dTHP1, differentiated THP1 cells; NQ, not quantifiable.

**
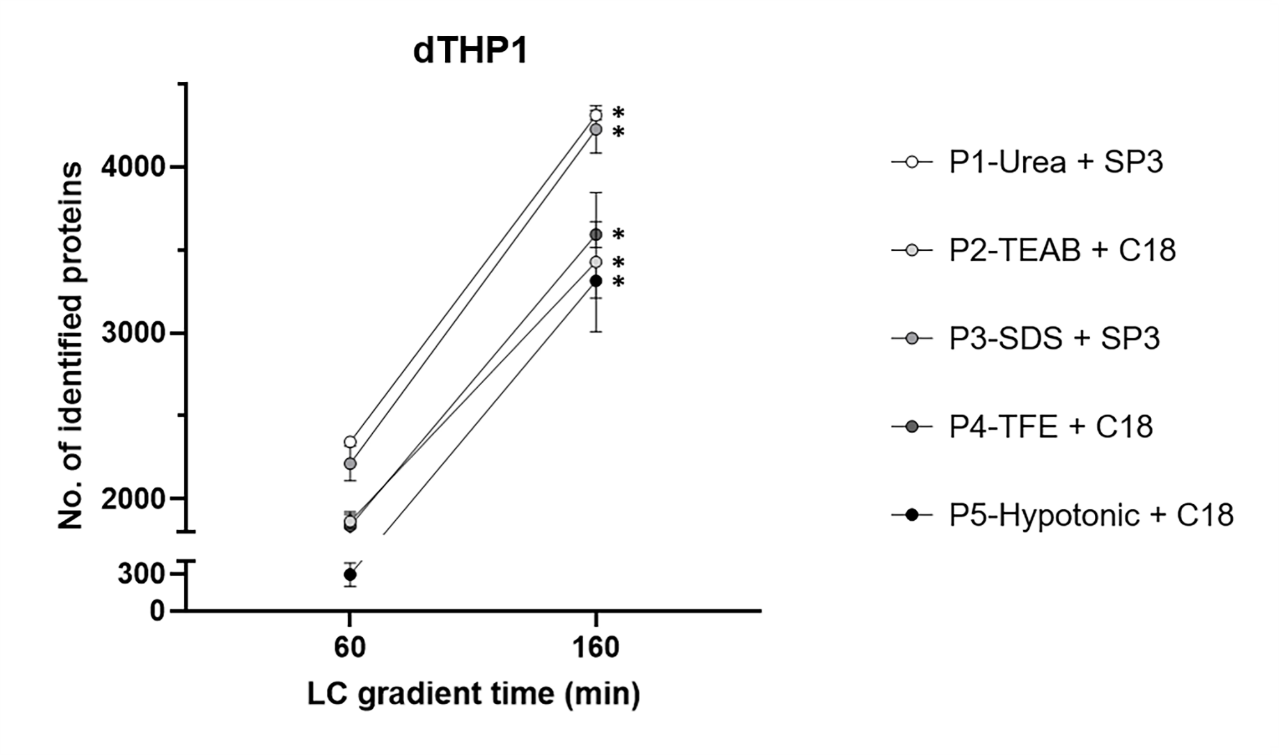
**

**Supplementary Figure S4.** Performance of short (60 min) and long (160 min) liquid chromatography (LC) gradient times on protein identification after lysis of 50,000 dTHP1 cells with different protocol combinations (P1-5 + C18/SP3). Each dot represents the median value for the replicates (including all identified proteins). * p-value <0.05 and 10% FDR vs 160 min after applying the Mann-Whitney test.

**
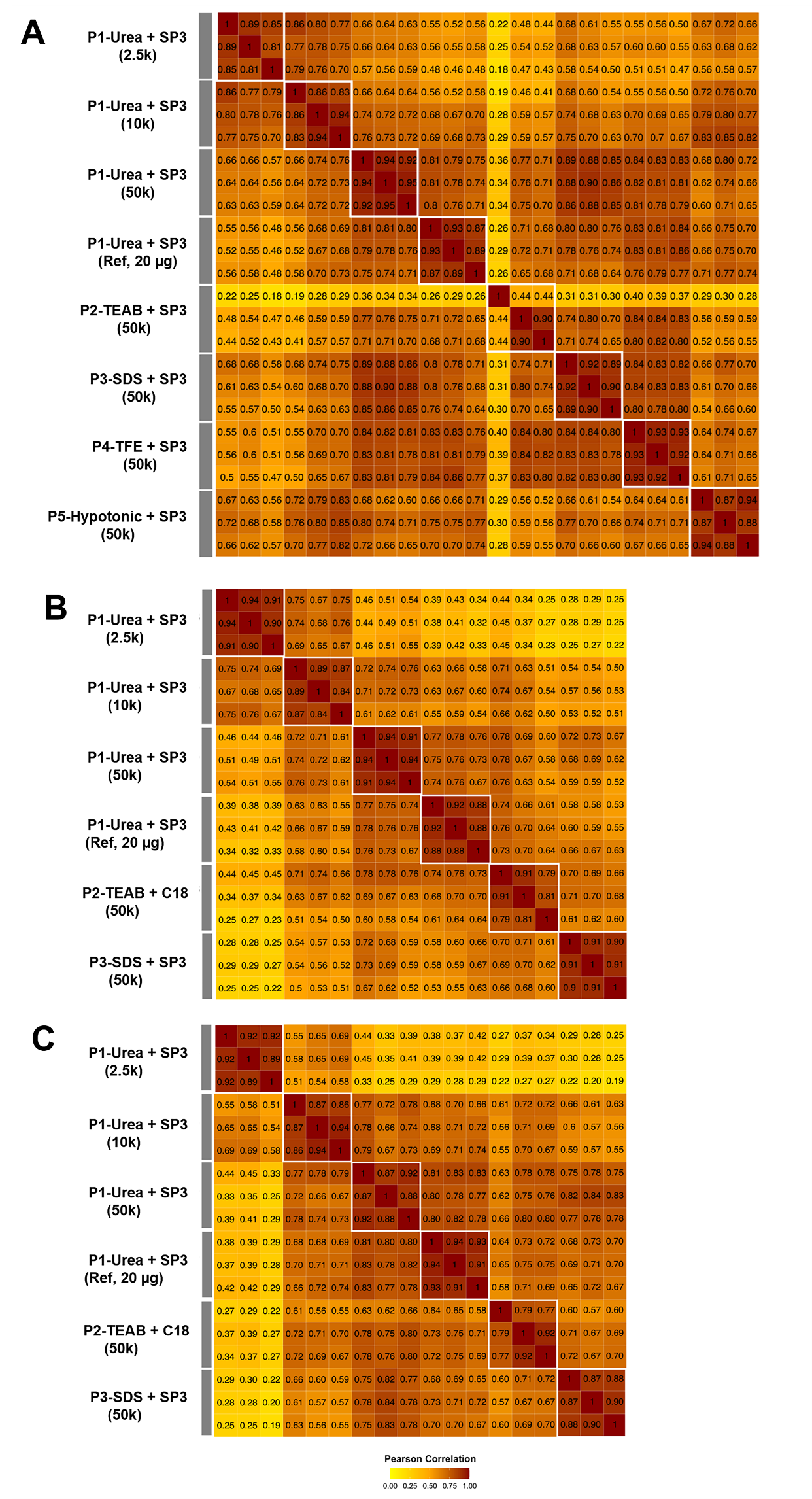
** **Supplementary Figure S5.** Graphs for the assessment of reproducibility. Pearson correlation R values were calculated for each pair-wise comparison by using the semi-quantitative proteomics data (Normalized Spectral Abundance Factor, NSAF) to evaluate the technical reproducibility of the three replicates (3x3 squares enclosed by a white line) performed per condition (i.e. protocol and cell number tested) measured using a long liquid chromatography gradient (i.e. 160 min). Also, the similarity of the different conditions can be inferred by evaluating their correlation values. A) dTHP1 cells; B) THP1 cells; C) HT-29 cells.


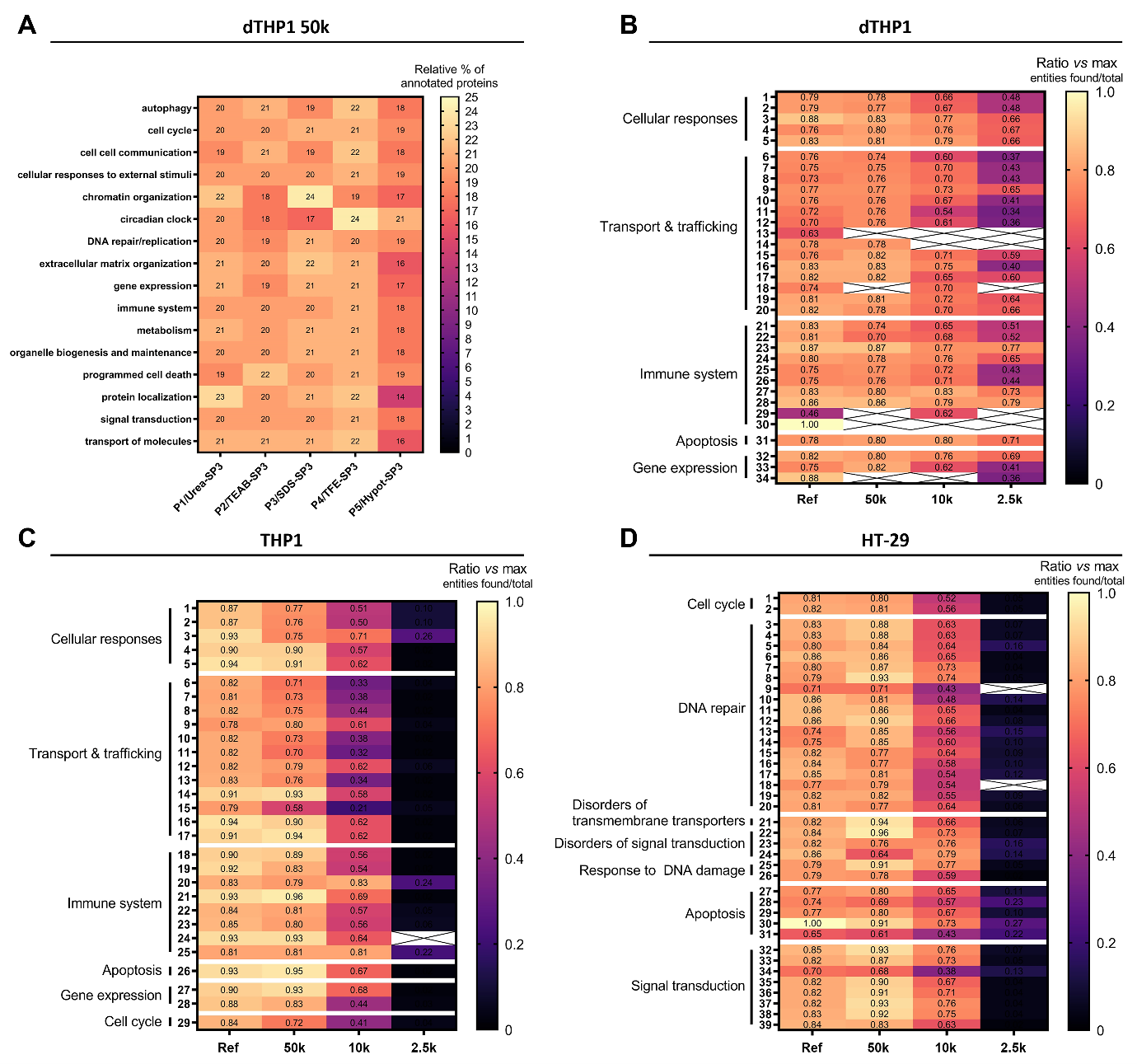


**Supplementary Figure S6.** Functional enrichment analysis of dTHP1, THP1 and HT-29 cells. A) Heatmap depicting the relative percentage (%) of annotated proteins from 50k dTHP1 cells in various protocols assigned to main pathways (from all identified proteins). B-D) Entities found/total entities ratios of significantly enriched pathways vs maximum, including proteins identified with ≥ 2 unique peptides from various cell numbers of B) dTHP1, C) THP1 and D) HT-29. Maximum refers to all proteins per pathway detected across REF, 50k, 10k and 2.5k cell samples; REF, a sample containing 20 µg of protein. Identified proteins were grouped based on their function using the Reactome pathway database (https://reactome.org). Lists of pathways shown in panels B-D are collected in Supplementary Table S6.

**
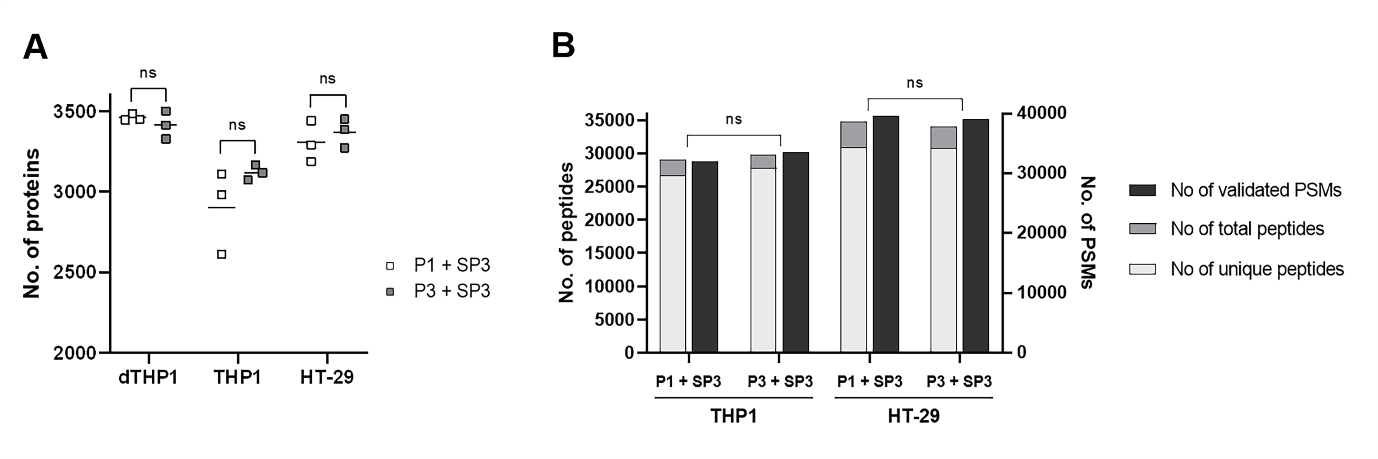
**

**Supplementary Figure S7.** Comparison of P1/urea and P3/SDS protocols combined with SP3 for the identification of proteins (A) and peptides/peptide spectrum matches (PSMs) (B) from 50,000 dTHP1, THP1 and HT-29 cells. Proteins were identified with at least two unique peptides per protein. ns, not significant. Statistical significance was determined with Mann Whitney test and Bonferroni-Dunn method to correct for multiple comparisons.


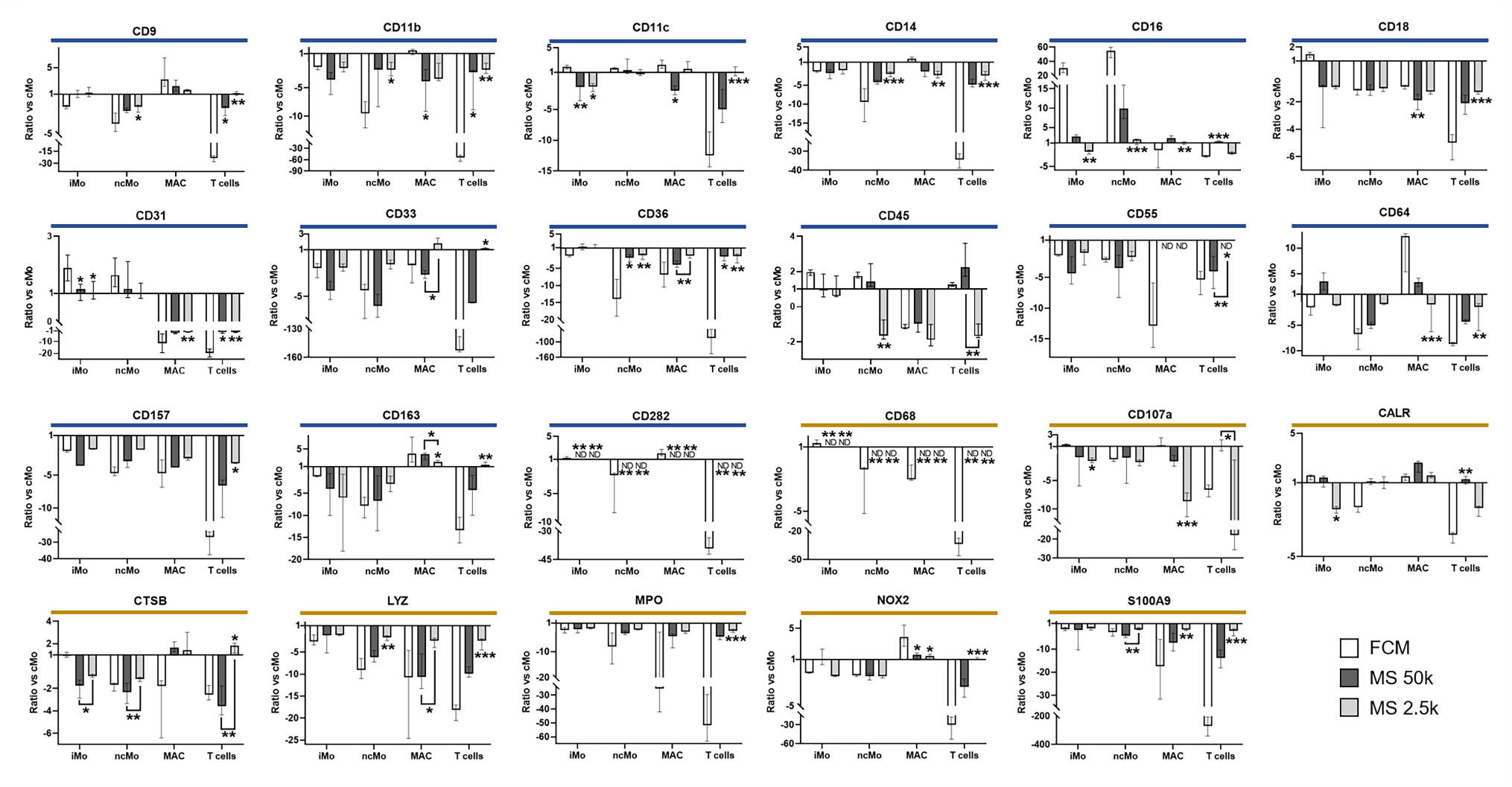


**Supplementary Figure S8.** Marker expression assessment by mass spectrometry(MS)-based vs flow cytometry (FCM) techniques. Expression ratios were calculated vs cMo for intermediate monocytes (iMo), non-classical monocytes (ncMo), and T cells from peripheral blood and macrophages/microglia (MAC) from glioblastomas samples by using the normalized abundance values (for MS) from 50k and 2.5k cells and the Mean Fluorescent Intensity (MFI, for FCM). Membrane-bound proteins are indicated in blue, whereas cytoplasmic proteins are underlined in yellow. Statistically significant differences were calculated using a Kruskal-Wallis test and Dunn’s method to correct for multiple comparisons (p-values: * ≤ 0.05; ** ≤ 0.01; *** ≤ 0.001). CALR, calreticulin; CTSB, cathepsin B; LYZ, lysozyme; MPO, myeloperoxidase.
