# Supplementary material for: Quantitative proteomics of small numbers of closely-related cells: Selection of the optimal method for a clinical setting
Source: Front Med (Lausanne). 2022 Sep 27;9:997305. doi: 10.3389/fmed.2022.997305 (PMC9553008; doi:10.3389/fmed.2022.997305)
Supplement: Supplementary file 1 [file Data_Sheet_1.zip › 997305_Supplementary Material/Supplementary Materials and Methods.docx]

**Supplementary Material**

**Quantitative proteomics of small numbers of closely-related cells: Selection of the optimal method for a clinical setting**

Kyra van der Pan^1^, Sara Kassem^1^, Indu Khatri^1,2^, Arnoud H de Ru^3^, George MC Janssen^3^, Rayman TN Tjokrodirijo^3^, Fadi al Makindji^1^, Eftychia Stavrakaki^4^, Anniek L de Jager^1^, Brigitta AE Naber^1^, Inge F de Laat^1^, Alesha Louis^1^, Wouter BL van den Bossche^4^, Lisette B Vogelezang^4^, Rutger K Balvers^4^, Martine LM Lamfers^4^, Peter A van Veelen^3^, Alberto Orfao^5^, Jacques JM van Dongen^1,5^, Cristina Teodosio^1,5†^, Paula Díez^1,5†^

^1^ Department of Immunology, Leiden University Medical Center (LUMC), Leiden, The Netherlands

^2^ Leiden Computational Biology Center, LUMC, Leiden, The Netherlands

^3^ Center for Proteomics and Metabolomics, LUMC, Leiden, The Netherlands

^4^ Department of Neurosurgery, Erasmus MC, Rotterdam, The Netherlands

^5^ Translational and Clinical Research Program, Cancer Research Center (IBMCC; University of Salamanca - CSIC); Cytometry Service, NUCLEUS; Department of Medicine, University of Salamanca and Institute of Biomedical Research of Salamanca (IBSAL), Spain

† These authors share last authorship

**Correspondence:** Prof. J.J.M van Dongen, MD, PhD

Leiden University Medical Center (LUMC)

J.J.M.van_Dongen@lumc.nl

**Supplementary Experimental Procedures**

**Peripheral blood (PB) processing**

### PB mononuclear cells (PBMCs) were collected from PB samples by Ficoll-Paque (Sigma-Aldrich, St. Louis, MO/US) gradient centrifugation, following the manufacturer’s instructions. Cell count was performed using an automated hematological analyser (Sysmex XP-300, Sysmex Europe GmbH, Norderstedt, Germany).

### **Sample collection and processing of tissues for validation of the method**

Bone marrow (BM) samples were obtained through BM aspiration from nine donors (no information on age and gender). BM mononuclear cells (MNC) were isolated by Ficoll-Paque gradient centrifugation, following the manufacturer´s instructions.

A total of 14 freshly obtained whole blood sodium citrate anticoagulated peripheral blood (PB) samples (median age 30, range 21-62, 5:9 female:male ratio) and three buffy coats (no information on age and gender) were collected at the Sanquin Blood Bank (Amsterdam, The Netherlands) and processed to obtain PBMCs as previously indicated. Seven whole blood samples were employed for *ex vivo*  isolation of myeloid antigen presenting cells, while the remaining 7 were stored for future *in vitro* stimulation experiments. The buffy coat samples were used for *in vitro* monocyte-derived macrophage differentiation and polarization assays. The samples employed for *in vitro* assays were stored in liquid nitrogen in RPMI containing 40% FCS and 10% dimethyl sulfoxide (DMSO) until further use. Prior to *in vitro*  cell culture, samples were thawed in cold PBS/1% BSA/ 10% FCS and further centrifuged for 5 min at 500g.

Skin cells were obtained from seven donors (no information on age and gender) after abdominoplastic surgery at Slotervaart Medical Center (Amsterdam, The Netherlands) and Medisch Facilitair Instituut Vlaardingen (Vlaardingen, The Netherlands). For each donor sample, 20-51 biopsies (6 mm diameter each) were taken using a biopsy punch (Cemex Trescon, Bleiswijk, The Netherlands) and processed using the Whole Skin Dissociation Kit (Miltenyi Biotec, Bergisch Gladbag, Germany) according to manufacturer’s instructions with slight modifications. Shortly, biopsies were incubated overnight at 37°C with enzymes A and D (enzyme P was not included due to epitope stripping) and pooled afterwards for further processing, as previously described (1, 2).

Peritoneal dialysate (PD) samples were collected at Leiden University Medical Center (LUMC, Leiden, The Netherlands) from six donors (median age 67, range 58 – 82, all male) undergoing peritoneal dialysis. Cell pellets were obtained upon centrifugation at 540 *g* for 5 min and resuspended in PBS.

Colon samples were obtained from seven colorectal cancer patients (unknown age and gender) after hemicolectomy surgery at LUMC. Tumoral and normal colon tissue pieces were immediately excised by pathologists and collected in cell medium (cold RPMI-1640 without phenol red (Lonza, Basel, Switzerland) supplemented with 20% FCS, 100 U/mL penicillin (Gibco, Gaithersburg, MD/US) and 100 µg/mL streptomycin (Gibco)). Within 30 min after collection, samples were cut into small pieces and incubated in a cell medium supplemented with 1 mg/mL collagenase D (Sigma) and 50 µg/mL of DNase (Sigma) at 37 °C for 15 min under rotation. After incubation, tissue pieces were dissociated on a gentleMACS™ Dissociator (Miltenyi Biotec). Both incubation and dissociation steps were repeated and obtained cell suspensions were filtered over a pre-wetted 70 µm cell strainer. To remove erythrocytes, cell samples were lysed in 1x NH_4_Cl (8.3 mg/mL NH_4_Cl and 1 mg/mL KHCO_3_ supplemented with 0.5 M EDTA) and subsequently washed twice with PBS/0.5% BSA/2 mM EDTA.

All samples were freshly processed within 4 h after collection. For tissues requiring an enzymatic digestion step (i.e. skin and colon), different methods and enzyme combinations were tested to ensure optimal cell yield while preserving protein epitopes for further immunophenotyping analysis (data not shown). In all cases, informed consent was provided according to the Declaration of Helsinki, guidelines of the local ethics committees and review boards (BM: 2018.IMM.BM; PB and buffy coats: NVT0532.01, Sanquin Blood Bank (Amsterdam, The Netherlands); skin: B18.009; PD: W2017.031; colon: NL66875.056.18).

### **Monocyte and dendritic cell (DC) enrichment**

PBMCs were incubated with biotin-labelled antibodies against CD3, CD15, CD19 and CD56 proteins (for exclusion of T cells, granulocytes, B cells and natural killer cells, respectively) (Supplementary Table S1, panel G) for 30 min at RT, and washed with PBS/0.5% BSA/2 mM EDTA. Cells were then incubated in Dynabeads® Biotin Binder (Thermo Fischer) for 30 min at 4 °C under continuous gentle rotation and placed on a BD IMag™ Cell Separation Magnet (BD Biosciences), washed and counted on a Sysmex hematological analyzer. Monocyte- and DC-enriched fraction was consecutively processed for sorting.

**Glioblastoma (GBM) processing**

Fresh GBM samples were collected from surgically resected tumor tissues and tumor aspirates were obtained using a cavitational ultrasonic surgical aspirator (CUSA)(3). Resected tumor tissues were collected in Dulbecco's Modified Eagle's Medium (DMEM) (Gibco, Gaithersburg, MD/US), supplemented with 100 U/mL penicillin, 100 μg/mL streptomycin (Thermo Fisher, Waltham, MA/USA). Samples were processed immediately after collection from the operating theatre as described earlier(4). Briefly, tumor tissues were firstly mechanically dissociated into small pieces with a scalpel. The tumor pieces were then enzymatically digested with 2 mg/mL collagenase A (Sigma-Aldrich) and 0.15 mg/mL DNase I (Sigma-Aldrich) in a dissociation medium (DMEM supplemented with 1% penicillin/streptomycin), by incubation at 37 °C for 15-40 min while shaking. The dissociated cells were then filtered through a 70 µm cell strainer, washed in PBS and incubated in erythrocyte lysis buffer (155 mM NH_4_Cl, 0.1 mM EDTA and 10 mM KHCO_3_ in Milli-Q water) for 10 min at 4 °C to remove the red blood cells. Subsequently, cells were washed and resuspended in Neurosphere (NS) medium, consisting of DMEM-F12 medium supplemented with 1% penicillin/streptomycin, 20 ng/mL basic Fibroblast Growth Factor (bFGF; Gibco), 20 ng/mL Epidermal Growth Factor (EGF; Gibco), 5 µg/mL heparin (Alfa Aesar, Haverhill, MA/US) and 2% B27 Supplement (50x) without vitamin A (Gibco). The tumor pieces that were collected with CUSA device were carefully scraped from the collection container and then processed as the tumor tissue pieces. Cells from surgically resected tumor tissues and CUSA tumor aspirates from the same patient were pooled, counted manually and stored at 4 ºC in NS medium until further processing.

### **Sorting of cell populations used for validation of the method**

Cell suspension containing BM MNC, monocyte- and DC-enriched PBMCs, skin, colon and PD cells were stained with fluorochrome-labelled monoclonal antibodies (Supplementary Table S1) for 30 min at RT, according to EuroFlow guidelines (www.EuroFlow.org). Upon staining, all samples were washed with PBS/0.5% BSA/2 mM EDTA, except for skin cells which were washed with PBS without any protein additive and further incubated with the LIVE/DEAD™ Fixable Aqua Dead Cell Stain Kit (Thermo Fischer) for 30 min at RT protected from light. A final wash with PBS was performed on all samples before sorting populations of interest on a BD FACSAria™ III cell sorter (BD Biosciences) according to sorting panels described in Supplementary Table S1. Samples were kept at 4 °C during sorting and collected in RPMI/ 10% FCS/1% protease/phosphatase inhibitor cocktails (PIC) and were further washed three times with ice-cold PBS/1% PIC (5 min, 1000 g, 4 °C) and cell pellets were freeze-dried and stored at -80 °C until further processing.

The samples used in subsequent in vitro studies (cMo, myDC, and pDC) were collected in the abovementioned collection medium without PIC and washed with RPMI/10% FCS/100 U/mL penicillin, 100 μg/mL streptomycin and 1% GlutaMAX™, prior to culture.

### **In vitro stimulation of monocytes and DC**

Collected cells (cMo, myDC and pDC) were then plated in 96-wells plates at a density of 0.03x10^6^ cells/well. cMo and myDC were incubated in 100 ng/mL LPS (Sigma-Aldrich), whereas pDC were incubated in 5µM CpG (InvivoGen, San Diego, CA, USA), to activate the cell via TLR4 and TLR9 signalling, respectively. All cell types were also incubated with 100 ng/mL R484 (InvivoGen) for activation via TLR7/8. After the addition of the stimuli, cells were incubated at 37 °C for 18 h. Unstimulated cells were taken along as controls. Cells were detached using trypsin-EDTA (Sigma-Aldrich), centrifuged for 5 min at 500g, washed three times with ice-cold PBS/1% PIC (5 min, 1000 g, 4 °C), freeze-dried and stored at -80 °C.

### **Monocyte-derived macrophage differentiation and polarization**

For monocyte-derived macrophages differentiation and polarization, isolation of PB CD14^+^ monocytes was performed prior to cell culture. Briefly, PBMCs were resuspended in degassed MACS buffer (PBS/0.5% BSA/2 mM EDTA) and incubated with CD14 Microbeads (Miltenyi-Biotec) at a ratio of 100:1 (v/v) cells:beads at 4 °C for 20 min, following the manufacturer´s instructions. Cells were further washed with MACS buffer prior to transfer to the LS column (Miltenyi-Biotec). The positive fraction was frozen in RPMI/40% FCS/10% DMSO and stored in liquid nitrogen.

For the differentiation assays, CD14^+^ cells were thawed in cold IMDM (Thermo Fischer) (supplemented with 10% FCS and 2% Penicillin/Streptomycin) and centrifuged for 5 min at 500g. Cells were washed once with IMDM and resuspended in IMDM at a concentration of 0.133x10^6^/mL. Cells were then incubated at 37 °C for 4 days in 5 ng/mL macrophage colony-stimulating factor (M-CSF; R&D Systems, Minneapolis, MN, USA). For macrophage polarization, after 4 days, the medium was replaced and cells were incubated an additional 24 h at 37 °C in the presence of either nothing (no stimulation), 50 ng/mL interferon- γ (IFN-γ; R&D Systems) or 50 ng/mL interleukin 4 (IL4; R&D Systems), to induce differentiation into M1 and M2 macrophages, respectively. Subsequently, cells were washed with PBS, detached using trypsin-EDTA, centrifuge for 5 min at 500g, washed three times with ice-cold PBS containing 1% PIC (5 min, 1000g, 4 °C), freeze-dried and stored at -80 °C.

**P5 Hypotonic lysis buffer: Details on definition of lysis solution composition**

For the P5 method, we aimed at using a very simple but effective hypotonic lysis buffer, skipping the usage of any detergent (as SDS) or chaotropic agent (as urea) as those reagents were already considered in P1-P4 lysis methods. After a literature search, we found there is no consensus on the composition of this type of hypotonic lysis solutions. Therefore, we defined our lysis recipe based on our previous experience and knowledge. Specifically, we included 30 mM HEPES as buffer, at pH 7.4, to avoid unnecessary pH changes. DTT was added as a reducing agent to prevent oxidative effects at 0.5 mM concentration since it is known that at 0.1 – 1 mM it does not form mixed disulfides with proteins (as β-mercaptoethanol does, for instance). 0.1 mM EDTA was included to chelate heavy metal ions that could affect the protein and promote oxidation, and 1% protease/phosphatase inhibitors to avoid protein degradation or modifications. Although salts could improve protein extraction, we did not include them since purification or analytical steps might be compromised. Additionally, freeze-thaw cycles of the sample were applied as non-mechanical forces to help with the disruption of the cell. This method was preferred due to its simplicity and accessibility (in contrast to e.g. ultrasonication which requires an ultrasonicator machine). Finally, all steps were performed in the same single tube, avoiding sample transfers that could result in protein losses and would be critical when working with low cell numbers.

**Protein quantification and silver staining**

Extracted proteins were quantified using the Qubit™ Protein Assay Kit (Thermo Fisher) on the Qubit™ 3.0 Fluorometer (Thermo Fisher) according to the manufacturer’s protocol. Overall, 1 μL/sample was separated on a 12% SDS-PAGE gel and silver stained using the Pierce™ Silver Stain Kit (Thermo Fisher) according to the manufacturer’s instructions.

**Sample preparation for mass spectrometry (MS) analysis**

Specific conditions for the reduction, alkylation and digestion of proteins obtained after cell lysis with P1-P5 protocols are indicated in Table 1.

Table 1. Sample preparation conditions for LC-MS/MS analysis.

| Processing strategy | Reduction (10 mM DTT) (incubation time, incubation temperature) | Alkylation  (IAA concentration, incubation time, incubation temperature) | Protein digestion  (enzyme:protein (w/w) ratio) | |
| --- | --- | --- | --- | --- |
|  |  |  | **Lys-C** | **Trypsin** |
| P1 (+SP3 or C18) | 30 min, 45°C | 40 mM, 30 min, RT | 1:75 | 1:25 |
| P2 (+SP3 or C18) | 30 min, RT | 50 mM, 30 min, RT | 1:50 | 1:50 |
| P3 (+SP3 or C18) | 30 min, 45°C | 40 mM, 30 min, RT | - | 1:40 |
| P4 (+SP3 or C18) | 60 min, RT | 25 mM, 30 min, RT | 1:50 | 1:50 |
| P5 (+SP3 or C18) | 45 min, RT | 25 mM, 30 min, RT | 1:100 | 1:50 |

*DTT*, dithiothreitol; *IAA*, iodoacetamide; *P*, protein extraction procedure; *RT*, room temperature; *SP3,* single-pot solid-phase-enhanced sample preparation.

**LC-MS/MS analysis for technical evaluation on cell lines**

An on-line C18 nano-High-Performance Liquid Chromatography (HPLC) MS/MS with a system consisting of an Easy nLC 1200 gradient HPLC system (Thermo Fisher) coupled to an Orbitrap Fusion™ Lumos™ Tribrid™ mass spectrometer (Thermo Fisher) was used for LC-MS/MS analysis of dTHP1, HT-29 and THP1 cell samples. Peptides were dissolved in 100/0.1 (v/v) water/formic acid (FA), loaded onto a homemade precolumn (100 μm x 15 mm; Reprosil-Pur C18-AQ 3 μm, Dr Maisch, Ammerbuch, Germany) and eluted via a homemade analytical nano-HPLC column (50 cm x 75 μm; ReproSil-Pur C18-AQ 1.9 μm). The gradient was run from 2% to 36% solvent B (20/80/0.1 (v/v/v) water/acetonitrile (ACN)/FA) in 40, 60 or 160 min. The nano-HPLC column was drawn to a tip of ∼5 μm which acted as the electrospray needle of the MS source. The Easy nLC-Orbitrap Fusion Lumos was operated in data-dependent MS/MS mode for a cycle time of 3 sec, with an HCD collision energy at 32 V and recording of the MS2 spectrum in the Orbitrap. Master scans (MS1) were acquired in the mass range of m/z 400-1500 with a 120,000 resolution, at an AGC target of 400,000 at a maximum fill time of 50 msec. Dynamic exclusion after n=1 was enabled for 60 sec, including charge states from 2 to 4. MS2 precursors were isolated via the quadrupole with an isolation width of 1.2 Da (first mass=110 Da). The MS2 scan resolution was 30,000 with an AGC target of 50,000 at a maximum fill time of 60 msec.

**TMT-labelled peptides LC-MS/MS analysis of monocytes from PB and GBM macrophages/microglia**

TMT-labelled peptides were dissolved in water/FA (100/0.1 v/v; solvent A) and subsequently analyzed by on-line C18 nano-HPLC MS/MS with a system consisting of an Ultimate3000 gradient HPLC system (Thermo Fisher), and an Exploris480 Orbitrap mass spectrometer (Thermo Fisher). Fractions (6 per TMT experiment) were injected onto a pre-column (300 μm × 5 mm; Pepmap100 C18 5 μm) cartridge (Thermo Fisher) and eluted via a homemade analytical nano-HPLC column (50 cm × 75 μm; ReproSil-Pur C18-AQ 1.9 µm). The analytical column temperature was maintained at 50 °C with a Sonation PRSO-V2 column oven. The gradient was run from 5% to 30% solvent B (20/80/0.1 (v/v/v/) water/ACN/FA) in 160 min. The nano-HPLC column was drawn to a tip of ∼5 μm and acted as the electrospray needle of the MS source. The Exploris480 mass spectrometer was set to use the TMT method. The MS1 spectrum was recorded in the Orbitrap (resolution 120,000; mass range 350−1600 m/z; automatic gain control (AGC) was set to standard; the maximum injection time was 50 msec). Dynamic exclusion was after n=1 with an exclusion duration of 45 sec and a mass tolerance of 10 ppm. Charge states 2-5 were included. Precursors for MS2 analysis were selected using a TopSpeed method of 3 sec at a resolution of 30,000. MS2 was performed by HCD at an NCE of 35 analysis; the AGC was set to custom at 200; the maximum injection time was set to auto. The isolation window for MS/MS was 1.2 Da.

**REFERENCES**

1. Winkel BMF, de Korne CM, van Oosterom MN, Staphorst D, Meijhuis M, Baalbergen E, et al. Quantification of wild-type and radiation attenuated Plasmodium falciparum sporozoite motility in human skin. Scientific reports. 2019;9(1):13436.

2. Winkel BMF, Pelgrom LR, van Schuijlenburg R, Baalbergen E, Ganesh MS, Gerritsma H, et al. Plasmodium sporozoites induce regulatory macrophages. PLoS Pathog. 2020;16(9):e1008799.

3. Schroeteler J, Reeker R, Suero Molina E, Brokinkel B, Holling M, Grauer OM, et al. Glioma tissue obtained by modern ultrasonic aspiration with a simple sterile suction trap for primary cell culture and pathological evaluation. Eur Surg Res. 2014;53(1-4):37-42.

4. Balvers RK, Kleijn A, Kloezeman JJ, French PJ, Kremer A, van den Bent MJ, et al. Serum-free culture success of glial tumors is related to specific molecular profiles and expression of extracellular matrix-associated gene modules. Neuro Oncol. 2013;15(12):1684-95.

5. Budnik B, Levy E, Harmange G, Slavov N. SCoPE-MS: mass spectrometry of single mammalian cells quantifies proteome heterogeneity during cell differentiation. Genome Biology. 2018;19(1):161.

6. Zhu Y, Piehowski PD, Zhao R, Chen J, Shen Y, Moore RJ, et al. Nanodroplet processing platform for deep and quantitative proteome profiling of 10-100 mammalian cells. Nature communications. 2018;9(1):882.

7. Li ZY, Huang M, Wang XK, Zhu Y, Li JS, Wong CCL, et al. Nanoliter-Scale Oil-Air-Droplet Chip-Based Single Cell Proteomic Analysis. Analytical Chemistry. 2018;90(8):5430-8.

8. Lou Q, Ma Y, Zhao SP, Du GS, Fang Q. A flexible and cost-effective manual droplet operation platform for miniaturized cell assays and single cell analysis. Talanta. 2021;224:121874.
