# Supplementary material for: Quantitative proteomics of small numbers of closely-related cells: Selection of the optimal method for a clinical setting
Source: Front Med (Lausanne). 2022 Sep 27;9:997305. doi: 10.3389/fmed.2022.997305 (PMC9553008; doi:10.3389/fmed.2022.997305)
Supplement: Supplementary file 1 [file Data_Sheet_1.zip › 997305_Supplementary Material/Supplementary Table Legends.docx]

**Supplementary Material**

**Quantitative proteomics of small numbers of closely-related cells: Selection of the optimal method for a clinical setting**

Kyra van der Pan^1^, Sara Kassem^1^, Indu Khatri^1,2^, Arnoud H de Ru^3^, George MC Janssen^3^, Rayman TN Tjokrodirijo^3^, Fadi al Makindji^1^, Eftychia Stavrakaki^4^, Anniek L de Jager^1^, Brigitta AE Naber^1^, Inge F de Laat^1^, Alesha Louis^1^, Wouter BL van den Bossche^4^, Lisette B Vogelezang^4^, Rutger K Balvers^4^, Martine LM Lamfers^4^, Peter A van Veelen^3^, Alberto Orfao^5^, Jacques JM van Dongen^1,5^, Cristina Teodosio^1,5†^, Paula Díez^1,5†^

^1^ Department of Immunology, Leiden University Medical Center (LUMC), Leiden, The Netherlands

^2^ Leiden Computational Biology Center, LUMC, Leiden, The Netherlands

^3^ Center for Proteomics and Metabolomics, LUMC, Leiden, The Netherlands

^4^ Department of Neurosurgery, Erasmus MC, Rotterdam, The Netherlands

^5^ Translational and Clinical Research Program, Cancer Research Center (IBMCC; University of Salamanca - CSIC); Cytometry Service, NUCLEUS; Department of Medicine, University of Salamanca and Institute of Biomedical Research of Salamanca (IBSAL), Spain

† These authors share last authorship

**Correspondence:** Prof. J.J.M van Dongen, MD, PhD

Leiden University Medical Center (LUMC)

J.J.M.van_Dongen@lumc.nl

**SUPPLEMENTARY TABLE LEGENDS**

**Supplementary Table S1. List of fluorochrome-conjugated antibodies used for flow cytometry-based assays.** Per antibody, protein marker name, fluorochrome, antibody clone, antibody identifier and panel where the antibody was used are indicated.

**Supplementary Table S2. Overview of detected proteins in 50k dTHP1 cells after an HPLC gradient run of 60 min.** Five cell lysis buffers were compared (P1-P5) in combination with two sample clean-up methods (SP3 and C18). *dTHP1,* differentiated THP1*;* *P1*, urea-based lysis buffer; *P2*, TEAB-based lysis buffer; *P3,* SDS-based lysis buffer; *P4,* TFE-based lysis buffer; *P5,* hypotonic lysis buffer; *SP3,* single-pot solid phase-enhanced sample preparation; *C18,* C18-column-based sample clean-up method.

**Supplementary Table S3. Overview of detected proteins in different numbers of dTHP1 cells after an HPLC gradient run of 160 min.** Proteins were extracted from 2.5k, 10k and 50k dTHP1 cells with various cell lysis buffers (P1-P5) combined with two sample clean-up methods (SP3 and C18). Also, 20 µg of protein were analyzed with the P1-SP3 protocol as a reference for complete proteome coverage. *dTHP1,* differentiated THP1; *P1,* urea-based lysis buffer; *P2,* TEAB-based lysis buffer; *P3*, SDS-based lysis buffer; *P4*, TFE-based lysis buffer; *P5*, hypotonic lysis buffer; *SP3,* single-pot solid phase-enhanced sample preparation; *C18*, C18-column-based sample clean-up method.

**Supplementary Table S4. Detailed description of selected features for proteomics sample preparation methods.** **A)** Estimated time required to perform each of the P1-P5 lysis methods and the C18 and SP3 clean-up protocols. **B)** Estimated cost for processing 100 samples with each of the P1-P5 and C18/SP3 methodologies. Common steps performed in all cases (e.g. cell washing, protein quantification, protein labelling) are not included. **C)** Summary of different features (accessibility, ease of use, time, cost and reproducibility) for the herein tested methods combined with SP3 and published strategies for single-cell analysis.

**Supplementary Table S5. Subcellular location of proteins identified in each of the tested procedures combined with SP3.** Proteins were extracted from 50k dTHP1 cells using 5 different lysis buffers (P1-P5), followed by sample clean-up with SP3. All proteins unique for each of these procedures are listed. Also, the combination of all datasets to determine the complete dTHP1 proteome and their subcellular location are collected here. *dTHP1*, differentiated THP1; P1, urea-based lysis buffer; *P2*, TEAB-based lysis buffer; *P3*, SDS-based lysis buffer; *P4*, TFE-based lysis buffer; *P5*, hypotonic lysis buffer; *SP3*, single-pot solid phase-enhanced sample preparation.

**Supplementary** **Table S6. Functional annotation of proteins extracted from various amounts of dTHP1 cells, THP1 cells and HT-29 cells.** Proteins were extracted from 20ug, 50k, 10k and 2.5k cells. Proteins identified in all replicates with ≥2 unique peptides were used for functional enrichment analysis using the Reactome pathway database. Pathways significantly enriched (p-value <0.05) are depicted.

**Supplementary Table S7. Overview of detected proteins in different numbers of THP1 and HT-29 cells after an HPLC gradient run of 160 min.** Proteins were extracted from 2.5k, 10k and 50k THP1 and HT-29 cells with various cell lysis buffers (P1-P3) combined with the SP3 sample clean-up method. Also, 20 µg of protein was analyzed with the P1-SP3 protocol. *P1*, urea-based lysis buffer; *P3*, SDS-based lysis buffer; *SP3*, single-pot solid phase-enhanced sample preparation.

**Supplementary Table S8. Quantified TMT-labelled proteins in cell populations from peripheral blood (PB) (i.e. classical monocytes, cMo; intermediate monocytes, iMo; non-classical monocytes, ncMo; T cells) and glioblastoma samples (i.e. macrophage/microglia, MAC; T cells).** All proteins have at least 2 unique peptides. *Quantified proteins* tab collects normalized abundances per sample and abundance ratios. *Present in all donors* tab includes the lists of proteins quantified in all tested donors per population and the corresponding grouped abundance values.

**Supplementary Table S9.** Functional enrichment analysis of major monocytic cell populations (cMo, iMo, ncMo) from peripheral blood and macrophages/microglia from glioblastoma samples. Analysis was performed using the Reactome Pathway database (https://reactome.org).

**Supplementary Table S10.** **Comparative analysis of -omics platforms.** Identification of 15 membrane and 8 cytoplasmic protein markers was evaluated by flow cytometry (FC) and mass spectrometry (MS) in paired samples of 50k and 2.5k cells of monocytes (cMo, iMo, ncMo) and T cells from peripheral blood and macrophages/microglia (MAC) from glioblastomas.

**Supplementary Table S11. Evaluation of the feasibility of the P1/urea-SP3 method in other cell types.** Information on the number of cells processed, protein amount per cell, total protein amount per sample, as well as number of distinct proteins identified by mass spectrometry analysis are indicated.
