# Supplementary material for: Quantitative proteomics of small numbers of closely-related cells: Selection of the optimal method for a clinical setting
Source: Front Med (Lausanne). 2022 Sep 27;9:997305. doi: 10.3389/fmed.2022.997305 (PMC9553008; doi:10.3389/fmed.2022.997305)
Supplement: Supplementary file 1 [file Data_Sheet_1.zip › 997305_Supplementary Material/Supplementary Table S1.docx]

**Supplementary Material**

**Quantitative proteomics of small numbers of closely-related cells: Selection of the optimal method for a clinical setting**

Kyra van der Pan^1^, Sara Kassem^1^, Indu Khatri^1,2^, Arnoud H de Ru^3^, George MC Janssen^3^, Rayman TN Tjokrodirijo^3^, Fadi al Makindji^1^, Eftychia Stavrakaki^4^, Anniek L de Jager^1^, Brigitta AE Naber^1^, Inge F de Laat^1^, Alesha Louis^1^, Wouter BL van den Bossche^4^, Lisette B Vogelezang^4^, Rutger K Balvers^4^, Martine LM Lamfers^4^, Peter A van Veelen^3^, Alberto Orfao^5^, Jacques JM van Dongen^1,5^, Cristina Teodosio^1,5†^, Paula Díez^1,5†^

^1^ Department of Immunology, Leiden University Medical Center (LUMC), Leiden, The Netherlands

^2^ Leiden Computational Biology Center, LUMC, Leiden, The Netherlands

^3^ Center for Proteomics and Metabolomics, LUMC, Leiden, The Netherlands

^4^ Department of Neurosurgery, Erasmus MC, Rotterdam, The Netherlands

^5^ Translational and Clinical Research Program, Cancer Research Center (IBMCC; University of Salamanca - CSIC); Cytometry Service, NUCLEUS; Department of Medicine, University of Salamanca and Institute of Biomedical Research of Salamanca (IBSAL), Spain

† These authors share last authorship

**Correspondence:** Prof. J.J.M van Dongen, MD, PhD

Leiden University Medical Center (LUMC)

J.J.M.van_Dongen@lumc.nl

**Supplementary Table S1. List of fluorochrome-conjugated antibodies used for flow cytometry-based assays.** Per antibody, protein marker name, fluorochrome, antibody clone, antibody identifier and panel where the antibody was used are indicated.

| **Marker** | **Fluorochrome** | **Antibody clone** | **Antibody identifier** | **Panel** |
| --- | --- | --- | --- | --- |
| **CD1a** | AF700 | HI149 | BioLegend Cat# 300120, RRID:AB_528764 | I |
| **CD3** | AF700 | SK7 | BioLegend Cat# 344822, RRID:AB_2563420 | A, B |
|  | Biotin | UCHT1 | BD Biosciences Cat# 555331, RRID:AB_395738 | G |
| **CD5** | BV510 | UCHT2 | BD Biosciences Cat# 563380, RRID:AB_2744435 | H |
| **CD9** | PerCP eFluor710 | M-L13 | Thermo Fisher Scientific Cat# 46-0098-41, RRID:AB_2762455 | C |
| **CD11b** | BV750 | M1/70 | BioLegend Cat# 101267, RRID:AB_2810328 | C |
| **CD11c** | BV480 | B-ly6 | BD Biosciences Cat# 566184, RRID:AB_2739581 | C |
|  | BV650 | B-ly6 | BD Biosciences Cat# 563403, RRID:AB_2732048 | B, J, K |
| **CD14** | APC H7 | MφP9 | BD Biosciences Cat# 641394, RRID:AB_1645725 | A, J |
|  | PE CF594 | MφP9 | BD Biosciences Cat# 562335, RRID:AB_11153663 | B, H, I, K, L |
|  | Qdot 800 | TuK4 | Thermo Fisher Scientific Cat# Q10064, RRID:AB_2556449 | C, D, E |
| **CD15** | Biotin | HI98 | Thermo Fisher Scientific Cat# 13-0159-82, RRID:AB_466375 | G |
| **CD16** | BV510 | 3G8 | BD Biosciences Cat# 563829, RRID:AB_2744296 | L |
|  | APC H7 | 3G8 | BD Biosciences Cat# 560195, RRID:AB_1645466 | C, D, E, F, H |
|  | FITC | CLB-Fc-gran1 | Sanquin Cat# M1604, RRID:AB_2877690 | A, J, K |
| **CD18** | SB600 | 6.7 | Thermo Fisher Scientific Cat# 63-0189-42, RRID:AB_2734896 | D |
| **CD19** | Biotin | HIB19 | BD Biosciences Cat# 555411, RRID:AB_395811 | G |
|  | PerCP Cy5.5 | SJ25C1 | BD Biosciences Cat# 332780, RRID:AB_2868629 | F |
| **CD31** | e450 | WM59 | Thermo Fisher Scientific Cat# 48-0319-41, RRID:AB_10853015 | D |
| **CD33** | PE Cy7 | P67.6 | BD Biosciences Cat# 333952, RRID:AB_2713932 | A, B, C, D, E, F, H, I, J, K |
| **CD34** | APC Cy7 | 581 | BioLegend Cat# 343514, RRID:AB_1877168 | L |
| **CD35** | BV605 | E11 | BD Biosciences Cat# 744276, RRID:AB_2742114 | L |
| **CD36** | FITC | CLB-IVC7 | Sanquin, Cat# M1613, RRID:AB_1155309 | H, L |
|  | PerCP Cy5.5 | CLB-IVC7 | Immunostep Cat# 36PP5.52, RRID:AB_2848146 | C |
| **CD44** | AF700 | BJ18 | BioLegend Cat# 338814, RRID:AB_2715999 | K |
| **CD45** | AF532 | HI30 | Thermo Fisher Scientific Cat# 58-0459-42, RRID:AB_11218673 | C, D, E |
|  | AF700 | HI30 | BD Biosciences Cat# 560566, RRID:AB_1645452 | H, L |
|  | APC H7 | 2D1 | BD Biosciences Cat# 641417, RRID:AB_2800453 | B, I, K |
|  | PO | HI30 | Thermo Fisher Scientific Cat# MHCD4530, RRID:AB_10376143 | A |
|  | OC515 | GA90 | Cytognos Cat# CYT-45OC, RRID:AB_2848147 | F, J |
| **CD55** | PE Cy5 | IA10 | BD Biosciences Cat# 555695, RRID:AB_396046 | C |
| **CD56** | Biotin | B159 | BD Biosciences Cat# 555515, RRID:AB_395905 | G |
| **CD62L** | BV650 | DREG-56 | BioLegend Cat# 304834, RRID:AB_2562130 | H |
| **CD64** | AF700 | 10.1 | BD Biosciences Cat# 561188, RRID:AB_10612007 | F |
|  | BV510 | 10.1 | BioLegend Cat# 305027, RRID:AB_2562512 | C, D, E |
|  | BV605 | 10.1 | BioLegend Cat# 305034, RRID:AB_2566237 | J, K |
|  | PE | 10.1 | BD Biosciences Cat# 644385, RRID:AB_1727085 | B, L |
| **CD68** | BV421 | Y1/82A | BioLegend Cat# 333828, RRID:AB_2800882 | D |
| **CD107a (LAMP1)** | BV786 | H4A3 | BD Biosciences Cat# 563869, RRID:AB_2738458 | D |
| **CD117 (c-Kit)** | PE Cy7 | 104D2D1 | Beckman Coulter, Cat# B49221, RRID:AB_2877689 | L |
| **CD141 (BDCA-3)** | BV421 | 1A4 | BD Biosciences Cat# 565321, RRID:AB_2739180 | H, I |
|  | PE | AD5-14H12 | Miltenyi Biotec Cat# 130-113-318, RRID:AB_2726095 | F |
| **CD157** | SB436 | SY11B5 | Thermo Fisher Scientific Cat# 62-1579-41, RRID:AB_2762523 | C |
| **CD163** | BV650 | GHI/61 | BD Biosciences Cat# 563888, RRID:AB_2738468 | L |
|  | BV711 | GHI/61 | BD Biosciences Cat# 563889, RRID:AB_2738469 | C |
| **CD166** | BV421 | 3A6 | BD Biosciences Cat# 562936, RRID:AB_2737905 | K |
| **CD192 (CCR2)** | BV605 | K036C2 | BioLegend Cat# 357214, RRID:AB_2563876 | H |
| **CD207 (Langerin)** | PE | 2G3 | BD Biosciences Cat# 564727, RRID:AB_2738920 | I |
| **CD282 (TLR2)** | AF647 | TL2.1 | BioLegend Cat# 309714, RRID:AB_2271923 | D |
| **CD300e (IREM2)** | APC | UP-H2 | Immunostep Cat# IREM2A-100T, RRID:AB_11140615 | A, F, H, J, L |
| **CD303** | APC | AC144 | Miltenyi Biotec Cat# 130-113-190, RRID:AB_2726015 | F, H |
| **CD326 (EpCAM)** | APC | HEA-125 | Miltenyi Biotec Cat# 130-113-260, RRID:AB_2726061 | K |
| **Calreticulin** | PerCP | 1G6A7 | Novus Cat# NBP1-47518PCP, RRID:AB_2883966 | D |
| **CTSB** | PE | D1C7Y | Cell Signaling Technology Cat# 59355, RRID:AB_2799564 | D |
| **FcERI** | FITC | AER-37 | Thermo Fisher Scientific Cat# 11-5899-42, RRID:AB_10732835 | F |
|  | PE | AER-37 | Thermo Fisher Scientific Cat# 12-5899-42, RRID:AB_10804885 | H, J |
| **HLA-DR** | BV570 | L243 | BioLegend Cat# 307638, RRID:AB_2650882 | E |
|  | PacB | L243 | BioLegend Cat# 307624, RRID:AB_493665 | F, J, L |
|  | PerCP Cy5.5 | G46-6 | BD Biosciences Cat# 552764, RRID:AB_394453 | A, B, H, I |
|  | PE | L243 | BD Biosciences Cat# 347401, RRID:AB_2629277 | K |
| **Lysozyme** | FITC | LZ-2 | Thermo Fisher Scientific Cat# GIC207, RRID:AB_2536533 | C |
| **MPO** | e450 | MPO0455-8E6 | Thermo Fisher Scientific Cat# 48-1299-42, RRID:AB_10804768 | C |
| **Nox2** | PE | 7D5 | LSBio (LifeSpan) Cat# LS-C179420, RRID:AB_2883967 | C |
| **Slan** | PE | DD.1 | Miltenyi Biotec Cat# 130-119-867, RRID:AB_2784456 | H |
| **S100A9 (MRP14)** | APC | REA859 | Miltenyi Biotec Cat# 130-114-708, RRID:AB_2726769 | C |
| **LIVE/DEAD™ Fixable Aqua Dead Cell Stain Kit** | BV510 | N/A | Thermo Fisher Scientific Cat# L34957 | I |
| **7AAD** | PerCP Cy5.5 | N/A | BD Biosciences Cat# 559925, RRID:AB_2869266 | J, K, L |

*AF*, alexa fluor; *APC*, allophycocyanin; *BV*, brilliant violet; *Cy,* cyanine; *FITC*, fluorescein isothiocyanate; *H7,* hilite 7*; PE*, phycoerythrin; *PerCP,* peridinin-chlorophyll-protein; *PacB*, pacific blue; *PO,* pacific orange; *SB,* Super Bright*.*

*Panel A*, sorting of monocyte populations and T cells from peripheral blood (PB) samples; *panel B*, sorting of macrophages/microglia (MAC) and T cells from glioblastoma (GBM) samples; *panels C and D*, marker combinations for mass spectrometry data validation; and *panel E*, backbone markers for the identification of monocyte populations and MAC from PB and GBM samples, respectively; *panel F,* sorting of B cells, monocyte and dendritic cell populations for *in vitro* stimulation; *panel G*, biotin-labelled antibodies used for the enrichment of the monocyte/DC fraction of PBMCs; *panel H,* sorting of monocyte and DC populations from the monocyte/DC enriched fraction of PBMCs; *panel I,* sorting of macrophage and dendritic cell populations from skin samples; *panel J,* sorting of macrophages and myDC from peritoneal dialysate samples; *panel K,* sorting of macrophages and epithelial cell populations from colon samples; *panel L,* sorting of immature monocytic populations from bone marrow samples.
