# Supplementary material for: Quantitative proteomics of small numbers of closely-related cells: Selection of the optimal method for a clinical setting
Source: Front Med (Lausanne). 2022 Sep 27;9:997305. doi: 10.3389/fmed.2022.997305 (PMC9553008; doi:10.3389/fmed.2022.997305)
Supplement: Supplementary file 1 [file Data_Sheet_1.zip › 997305_Supplementary Material/Supplementary Table S4.docx]

**Supplementary Material**

**Quantitative proteomics of small numbers of closely-related cells: Selection of the optimal method for a clinical setting**

Kyra van der Pan^1^, Sara Kassem^1^, Indu Khatri^1,2^, Arnoud H de Ru^3^, George MC Janssen^3^, Rayman TN Tjokrodirijo^3^, Fadi al Makindji^1^, Eftychia Stavrakaki^4^, Anniek L de Jager^1^, Brigitta AE Naber^1^, Inge F de Laat^1^, Alesha Louis^1^, Wouter BL van den Bossche^4^, Lisette B Vogelezang^4^, Rutger K Balvers^4^, Martine LM Lamfers^4^, Peter A van Veelen^3^, Alberto Orfao^5^, Jacques JM van Dongen^1,5^, Cristina Teodosio^1,5†^, Paula Díez^1,5†^

^1^ Department of Immunology, Leiden University Medical Center (LUMC), Leiden, The Netherlands

^2^ Leiden Computational Biology Center, LUMC, Leiden, The Netherlands

^3^ Center for Proteomics and Metabolomics, LUMC, Leiden, The Netherlands

^4^ Department of Neurosurgery, Erasmus MC, Rotterdam, The Netherlands

^5^ Translational and Clinical Research Program, Cancer Research Center (IBMCC; University of Salamanca - CSIC); Cytometry Service, NUCLEUS; Department of Medicine, University of Salamanca and Institute of Biomedical Research of Salamanca (IBSAL), Spain

† These authors share last authorship

**Correspondence:** Prof. J.J.M van Dongen, MD, PhD

Leiden University Medical Center (LUMC)

J.J.M.van_Dongen@lumc.nl

**Supplementary Table S4. Detailed description of selected features for proteomics sample preparation methods.** **A)** Estimated time required to perform each of the P1-P5 lysis methods and the C18 and SP3 clean-up protocols. **B)** Estimated cost (Dutch pricing) for processing 100 samples with each of the P1-P5 and C18/SP3 methodologies. Common steps performed in all cases (e.g. cell washing, protein quantification, protein labelling) are not included. **C)** Summary of different features (accessibility, ease of use, time, cost and reproducibility) for the herein tested methods combined with SP3 and published strategies for single-cell analysis.

**A. Time**

| Lysis strategy | A. Time | | | | | |
| --- | --- | --- | --- | --- | --- | --- |
|  | **Preparation of solutions** | **Centrifugation/incubation steps** | **Reduction step** | **Alkylation step** | **Digestion step** | **TOTAL** |
| P1/urea | 5 min | 15 min | 30 min | 30 min | 16 h | 17 h 20 min |
| P2/TEAB | 5 min | 12 h + 15 min | 30 min | 30 min | 16 h | 29 h 20 min |
| P3/SDS | 5 min | 5 min + 15 min | 30 min | 30 min | 12 h | 13 h 25 min |
| P4/TFE | 5 min | 120 min + 2 min | 60 min | 30 min | 16 h | 19 h 37 min |
| P5/hypotonic | 5 min | 5 min + 20 min | 45 min | 30 min | 16 h | 17 h 45 min |
| Clean-up strategy | **Preparation of bead solution/filters** | **Incubation/processing** **steps** | **TOTAL** | **Samples processed at a time (high throughput)** | | |
| SP3 | 5 min | 36 min + 26 min | 67 min | 1-96 ^a^ | | |
| C18 | 5 min | 5-20 min | 10-30 min | 1^b^ | | |

^a^ Depending on the magnet device used. Of note, when processing more samples, some extra time will be needed due to pipetting, but it is not significant (e.g. ~90 min total for 96 samples using a 96-well magnet plate).

^b^ Since each sample must be loaded and filtered individually over the column device, it is not possible to multiplex resulting in a significant increase in required time when processing a set of samples. Some time can be saved if a microcentrifuge is used during the filtration step (instead of manually operated), but still, there is a limitation of 24 samples per set and pipetting takes unavoidable time.

**B. Cost**

| Processing strategy | Cost (€) (for 100 samples, 50k cells per sample in 70 μl processing volume) | | | | | | |
| --- | --- | --- | --- | --- | --- | --- | --- |
|  | **Lysis buffer** | **Reduction buffer** | **Alkylation buffer** | **Digestion buffer** | **C18** | **SP3** | **TOTAL (€)** |
| P1/urea | 23.90 | 0.20 | 0.08 | 155.80 | 18.80 | - | **198.78** |
|  |  |  |  |  | - | 23.98 | **203.96** |
| P2/TEAB | 14.10 | 0.20 | 0.10 | 216.90 | 18.80 | - | **250.10** |
|  |  |  |  |  | - | 23.98 | **255.28** |
| P3/SDS | 29.40 | 0.20 | 0.08 | 105.00 | 18.80 | - | **153.48** |
|  |  |  |  |  | - | 23.98 | **158.66** |
| P4/TFE | 19.00 | 0.20 | 0.05 | 216.90 | 18.80 | - | **254.95** |
|  |  |  |  |  | - | 23.98 | **260.13** |
| P5/hypotonic | 23.50 | 0.20 | 0.05 | 112.60 | 18.80 | - | **155.15** |
|  |  |  |  |  | - | 23.98 | **160.33** |

**C. Summary of features**

| Strategy | Type of sample | Specific instrument requirements^a^ | Feature-based ranking of methods | | | | |
| --- | --- | --- | --- | --- | --- | --- | --- |
|  |  |  | **Accessibility**  (1: most accessible –  4: least accessible) | **Ease of use**  (1: easiest - 8: most difficult) | **Total processing time**  (1: shortest - 8: longest) | **Cost**  (1: cheapest – 8: most expensive) | **Reproducibility^b^** (1: most reproducible – 7: least reproducible) |
| P1/urea-SP3 | Paucicellular | Ultrasonicator bath, magnet | **1** | **1** | **2** | **3** | **1** |
| P2/TEAB-SP3 | Paucicellular | Ultrasonicator bath, magnet | **1** | **5** | **5** | **4** | **7** |
| P3/SDS-SP3 | Paucicellular | Heat thermoblock, magnet | **1** | **2** | **1** | **1** | **4** |
| P4/TFE-SP3 | Paucicellular | Incubator, ultrasonicator bath, magnet | **1** | **3** | **4** | **5** | **2** |
| P5/hypotonic-SP3 | Paucicellular | Heat thermoblock, magnet | **1** | **4** | **3** | **2** | **5** |
| SCoPE-MS | Single-cell | FACS sorter, focused ultrasonicator | **2** | **6** | **6** | **6** | **6** |
| nanoPOTS | Single-cell | Direct-write lithography system (for array printing), milling machine (for glass spacer), home-built liquid handling system, home-built program (to control liquid dispensing), Lexan chamber (to maintain humidity) | **4** | **8** | **8** | **8** | **3** |
| OAD chip/MDOS | Single-cell | Home-built liquid handling system ($40k for OAD system, $4k for MDOS) | **3** | **7** | **7** | **7** | **unknown** |

^a^ Common equipment generally available in the laboratories (e.g. microcentrifuges) is not included here.

^b^ Based on correlations between replicated measurements. For methods P1-P5, reproducibility values can be checked in Supplementary Figure S5A. For SCoPE-MS, a correlation between 60 and 85%, as described by Budnik B. *et al.* (5). For nanoPOTS, Pearson´s correlation from 0.91 to 0.94, as described by Zhu Y. *et al.* (6). For OAD/MDOS (7, 8), no information is given.

*SCoPE-MS,* Single Cell ProtEomics by Mass Spectrometry; *nanoPOTS,* nanodroplet Processing in One pot for Trace Samples; *OAD,* nanoliter-scale Oil-Air-Droplet; *MDOS,* Manual Droplet Operation System; *FACS,* Fluorescence-Activated Cell Sorting.
