# Supplementary material for: Quantitative proteomics of small numbers of closely-related cells: Selection of the optimal method for a clinical setting
Source: Front Med (Lausanne). 2022 Sep 27;9:997305. doi: 10.3389/fmed.2022.997305 (PMC9553008; doi:10.3389/fmed.2022.997305)
Supplement: Supplementary file 2 [file Data_Sheet_2.zip › 997305_Supplementary Material II/Supplementary Table S10.docx]

**Supplementary Material**

**Quantitative proteomics of small numbers of closely-related cells: Selection of the optimal method for a clinical setting**

Kyra van der Pan^1^, Sara Kassem^1^, Indu Khatri^1,2^, Arnoud H de Ru^3^, George MC Janssen^3^, Rayman TN Tjokrodirijo^3^, Fadi al Makindji^1^, Eftychia Stavrakaki^4^, Anniek L de Jager^1^, Brigitta AE Naber^1^, Inge F de Laat^1^, Alesha Louis^1^, Wouter BL van den Bossche^4^, Lisette B Vogelezang^4^, Rutger K Balvers^4^, Martine LM Lamfers^4^, Peter A van Veelen^3^, Alberto Orfao^5^, Jacques JM van Dongen^1,5^, Cristina Teodosio^1,5†^, Paula Díez^1,5†^

^1^ Department of Immunology, Leiden University Medical Center (LUMC), Leiden, The Netherlands

^2^ Leiden Computational Biology Center, LUMC, Leiden, The Netherlands

^3^ Center for Proteomics and Metabolomics, LUMC, Leiden, The Netherlands

^4^ Department of Neurosurgery, Erasmus MC, Rotterdam, The Netherlands

^5^ Translational and Clinical Research Program, Cancer Research Center (IBMCC; University of Salamanca - CSIC); Cytometry Service, NUCLEUS; Department of Medicine, University of Salamanca and Institute of Biomedical Research of Salamanca (IBSAL), Spain

† These authors share last authorship

**Correspondence:** Prof. J.J.M van Dongen, MD, PhD

Leiden University Medical Center (LUMC)

J.J.M.van_Dongen@lumc.nl

**Supplementary Table S10.** **Comparative analysis of -omics platforms.** Identification of 15 membrane and 8 cytoplasmic protein markers was evaluated by flow cytometry (FC) and mass spectrometry (MS) in paired samples of 50k and 2.5k cells of monocytes (cMo, iMo, ncMo) and T cells from peripheral blood and macrophages/microglia (MAC) from glioblastomas.

|  | | FC vs MS comparison  *n* detected by FC/ *n* detected by MS  (% of concordance) | | | | | | | | | | | | |
| --- | --- | --- | --- | --- | --- | --- | --- | --- | --- | --- | --- | --- | --- | --- |
|  | | **50k cells** | | | | | | **2.5k cells** | | | | | | |
| Marker | | **cMo** | **iMo*** | **ncMo** | **T cells** | **MAC*** | **Average (%)** | **cMo** | **iMo** | **ncMo** | **T cells** | **MAC*** | **Average (%)** |  |
| Membrane | **CD9** | 6/6  (100%) | 5/5  (100%) | 6/6  (100%) | 6/6  (100%) | 5/5  (100%) | 100 | 6/6  (100%) | 6/6  (100%) | 6/5  (83.3%) | 6/6  (100%) | 5/5  (100%) | 96.7 |  |
|  | **CD11b** | 6/6  (100%) | 5/5  (100%) | 6/6  (100%) | 1/6  (16.6%) | 5/5  (100%) | 83.3 | 6/6  (100%) | 6/6  (100%) | 6/6  (100%) | 1/6  (16.6%) | 5/5  (100%) | 83.3 |  |
|  | **CD11c** | 6/6  (100%) | 5/5  (100%) | 6/6  (100%) | N/A | 5/5  (100%) | 100 | 6/4  (66.6%) | 6/6  (100%) | 6/5  (83.3%) | N/A | 5/5  (100%) | 87.5 |  |
|  | **CD14** | 6/6  (100%) | 5/5  (100%) | 3/6  (50%) | N/A | 5/5  (100%) | 87.5 | 6/6  (100%) | 6/6  (100%) | 3/6  (50%) | N/A | 5/5  (100%) | 87.5 |  |
|  | **CD16** | 4/6  (66.6%) | 5/5  (100%) | 6/6  (100%) | N/A | 5/5  (100%) | 91.7 | 4/6  (66.6%) | 6/6  (100%) | 6/6  (100%) | N/A | 5/5  (100%) | 91.7 |  |
|  | **CD18** | 6/6  (100%) | 5/5  (100%) | 6/6  (100%) | 6/6  (100%) | 5/5  (100%) | 100 | 6/6  (100%) | 6/6  (100%) | 6/6  (100%) | 6/6  (100%) | 5/5  (100%) | 100 |  |
|  | **CD31** | 6/6  (100%) | 5/5  (100%) | 6/6  (100%) | 5/6  (83.3%) | 5/5  (100%) | 96.7 | 6/6  (100%) | 6/6  (100%) | 6/6  (100%) | 5/6  (83.3%) | 5/5  (100%) | 96.7 |  |
|  | **CD33** | 6/2  (33.3%) | 5/2  (40%) | 6/2  (33.3%) | N/A | 5/2  (40%) | 36.7 | 6/2  (33.3%) | 6/2  (33.3%) | 6/2  (33.3%) | N/A | 5/3  (60%) | 40.0 |  |
|  | **CD36** | 6/6  (100%) | 5/5  (100%) | 6/6  (100%) | 3/6  (50%) | 5/5  (100%) | 90.0 | 6/6  (100%) | 6/6  (100%) | 6/6  (100%) | 3/6  (50%) | 5/5  (100%) | 90.0 |  |
|  | **CD45** | 6/6  (100%) | 5/5  (100%) | 6/6  (100%) | 6/6  (100%) | 5/5  (100%) | 100 | 6/6  (100%) | 6/6  (100%) | 6/6  (100%) | 6/6  (100%) | 5/5  (100%) | 100 |  |
|  | **CD55** | 6/2  (33.3%) | 5/3  (60%) | 6/4  (66.6%) | 6/4  (66.6%) | 5/0  (0%) | 45.3 | 6/4  (66.6%) | 6/4  (66.6%) | 6/4  (66.6%) | 6/0  (0%) | 5/0  (0%) | 40.0 |  |
|  | **CD64** | 6/4  (66.6%) | 5/2  (40%) | 1/ 2  (50%) | N/A | 5/5  (100%) | 64.2 | 6/2  (33.3%) | 6/2  (33.3%) | 1/2  (50%) | N/A | 5/5  (100%) | 54.2 |  |
|  | **CD157** | 6/4  (66.6%) | 5/1  (20%) | 6/4  (66.6%) | 0/4  (0%) | 5/1  (20%) | 34.6 | 6/2  (33.3%) | 6/2  (33.3%) | 6/1  (16.6%) | 0/2  (0%) | 5/2  (33.3%) | 23.3 |  |
|  | **CD163** | 6/4  (66.6%) | 5/4  (80%) | 3/4  (75%) | N/A | 5/5  (100%) | 80.4 | 6/4  (66.6%) | 6/4  (66.6%) | 3/4  (75%) | N/A | 5/5  (100%) | 77.1 |  |
|  | **CD282**^#^ | 6/0  (0%) | 5/0  (0%) | 6/0  (0%) | 6/0  (0%) | 5/0  (0%) | 0 | 6/0  (0%) | 6/0  (0%) | 6/0  (0%) | 6/0  (0%) | 5/0  (0%) | 0 |  |
| Cytoplasmic | **CD68**^#^ | 6/0  (0%) | 5/0  (0%) | 6/0  (0%) | N/A | 5/0  (0%) | 0 | 6/0  (0%) | 6/0  (0%) | 6/0  (0%) | N/A | 5/0  (0%) | 0 |  |
|  | **CD107a** | 6/6  (100%) | 5/5  (100%) | 6/6  (100%) | 0/6  (0%) | 5/5  (100%) | 80.0 | 6/6  (100%) | 6/6  (100%) | 6/6  (100%) | 0/6  (0%) | 5/5  (100%) | 100 |  |
|  | **CALR** | 6/6  (100%) | 5/5  (100%) | 6/6  (100%) | 6/6  (100%) | 5/5  (100%) | 100 | 6/6  (100%) | 6/6  (100%) | 6/6  (100%) | 6/6  (100%) | 5/5  (100%) | 100 |  |
|  | **CTSB** | 6/6  (100%) | 5/5  (100%) | 1/6  (16.6%) | N/A | 3/5  (60%) | 69.2 | 6/6  (100%) | 6/6  (100%) | 1/6  (16.6%) | N/A | 3/5  (60%) | 69.2 |  |
|  | **LYZ** | 6/6  (100%) | 5/5  (100%) | 5/6  (83.3%) | N/A | 5/5  (100%) | 95.8 | 6/6  (100%) | 6/6  (100%) | 5/6  (83.3%) | N/A | 5/5  (100%) | 95.8 |  |
|  | **MPO** | 6/6  (100%) | 5/5  (100%) | 6/6  (100%) | N/A | 5/5  (100%) | 100 | 6/6  (100%) | 6/6  (100%) | 6/6  (100%) | N/A | 5/5  (100%) | 100 |  |
|  | **NOX2** | 6/6  (100%) | 5/5  (100%) | 6/6  (100%) | 0/6  (0%) | 5/5  (100%) | 80.0 | 6/6  (100%) | 6/6  (100%) | 6/6  (100%) | 0/6  (0%) | 5/5  (100%) | 100 |  |
|  | **S100A9** | 6/6  (100%) | 5/5  (100%) | 6/6  (100%) | N/A | 5/5  (100%) | 100 | 6/6  (100%) | 6/6  (100%) | 6/6  (100%) | N/A | 5/5  (100%) | 100 |  |
|  |  |  |  |  |  |  |  |  |  |  |  |  |  |  |

Six biological replicates per technique were evaluated per population, except for *samples, assessed in 5 donors. ^#^ CD68 and CD282 proteins were detected but not quantified by MS and no sample assignment was performed. *CALR,* calreticulin; *cMo*, classical monocytes; *CTSB,* cathepsin B; *FC,* flow cytometry; iMo*,* intermediate monocytes; *ncMo,* non-classical monocytes; *LYZ,* lysozyme C; *MAC,* glioblastoma macrophages/microglia; *MPO,* myeloperoxidase; *MS,* mass spectrometry; N/A; not applicable as marker is not expressed by the referred population.
