# Supplementary material for: Quantitative proteomics of small numbers of closely-related cells: Selection of the optimal method for a clinical setting
Source: Front Med (Lausanne). 2022 Sep 27;9:997305. doi: 10.3389/fmed.2022.997305 (PMC9553008; doi:10.3389/fmed.2022.997305)
Supplement: Supplementary file 2 [file Data_Sheet_2.zip › 997305_Supplementary Material II/Supplementary Table S11.docx]

**Supplementary Material**

**Quantitative proteomics of small numbers of closely-related cells: Selection of the optimal method for a clinical setting**

Kyra van der Pan^1^, Sara Kassem^1^, Indu Khatri^1,2^, Arnoud H de Ru^3^, George MC Janssen^3^, Rayman TN Tjokrodirijo^3^, Fadi al Makindji^1^, Eftychia Stavrakaki^4^, Anniek L de Jager^1^, Brigitta AE Naber^1^, Inge F de Laat^1^, Alesha Louis^1^, Wouter BL van den Bossche^4^, Lisette B Vogelezang^4^, Rutger K Balvers^4^, Martine LM Lamfers^4^, Peter A van Veelen^3^, Alberto Orfao^5^, Jacques JM van Dongen^1,5^, Cristina Teodosio^1,5†^, Paula Díez^1,5†^

^1^ Department of Immunology, Leiden University Medical Center (LUMC), Leiden, The Netherlands

^2^ Leiden Computational Biology Center, LUMC, Leiden, The Netherlands

^3^ Center for Proteomics and Metabolomics, LUMC, Leiden, The Netherlands

^4^ Department of Neurosurgery, Erasmus MC, Rotterdam, The Netherlands

^5^ Translational and Clinical Research Program, Cancer Research Center (IBMCC; University of Salamanca - CSIC); Cytometry Service, NUCLEUS; Department of Medicine, University of Salamanca and Institute of Biomedical Research of Salamanca (IBSAL), Spain

† These authors share last authorship

**Correspondence:** Prof. J.J.M van Dongen, MD, PhD

Leiden University Medical Center (LUMC)

J.J.M.van_Dongen@lumc.nl

**Supplementary Table S11. Evaluation of the feasibility of the P1/urea-SP3 method in other cell types.** Information on number of cells processed, protein amount per cell, total protein amount per sample, as well as number of distinct proteins identified by mass spectrometry analysis are indicated.

| **Tissue** | **Cell population** | **# Biological replicates** | **Cell number**  **[median (range)]** | **Protein (pg) per cell**  **[median (range)]** | **Total protein (μg) [median (range)]** | **# Proteins identified in at least 1 donor** | **# Proteins identified in all donors** |
| --- | --- | --- | --- | --- | --- | --- | --- |
| **Bone marrow** | Monoblast | 5 | 20,406 (2,100-65,490) | 230 (70 – 2,000) | 5 (4 – 5) | 3,564 | 2,199 |
|  | Promonocyte CD14^-^ | 5 | 40,796 (10,464-159,156) | 130 (30 – 400) | 5 (4 – 5) | 3,472 | 2,213 |
|  | Promonocyte CD14^dim^ | 5 | 75,812 (33,287-136,156) | 90 (70 – 150) | 7 (5 – 10) | 3,565 | 2,200 |
|  | Promonocyte CD14^+^ | 5 | 100,000 (32,061-100,027) | 80 (60 – 180) | 7 (6 – 9) | 3,440 | 2,322 |
|  | Mature cMo | 5 | 93,372 (61,448-112,189) | 80 (60 – 110) | 7 (6 – 10) | 3,505 | 2,271 |
| **Peripheral blood** | iMo | 4 | 10,768 (3,781-148,022) | 580 (100 – 1,080) | 4 (3 – 6) | 3,452 | 2,351 |
|  | myDC CD141^+^ | 5 | 8,911 (1,700-22,312) | 710 (230 – 2,330) | 4 (2 – 5) | 3,478 | 2,208 |
|  | myDC CD1c^+^ CD14^dim^ | 4 | 45,222 (11,008-72,967) | 150 (100 – 650) | 3 (2 – 7) | 3,514 | 2,290 |
|  | myDC CD1c^+^ CD14^-^ CD5^-^ | 4 | 15,276 (12,014-16,400) | 310 (270 – 480) | 5 (4 – 7) | 3,452 | 2,351 |
|  | myDC CD1c^+^ CD14^-^ CD5^+^ | 4 | 4,623 (263-9,602) | 1,360 (620 – 16,730) | 5 (4 – 9) | 3,502 | 2,279 |
|  | pDC | 4 | 9,837 (6,329-167,771) | 580 (80 – 800) | 5 (4 – 6) | 3,502 | 2,277 |
|  | B cells | 7 | 35,000 (35,000 – 35,000) | 117 (100 – 126) | 4 (4 - 4) | NA | NA |
| **Skin** | Macrophage | 5 | 66,886 (61,488-200,838) | 90 (80 – 110) | 7 (6 – 19) | 3,558 | 2,233 |
|  | DC CD1a^+^ | 5 | 43,006 (18,006-163,372) | 170 (80 – 1,700) | 6 (4 – 12) | 3,566 | 2,217 |
|  | DC CD14^+^ | 5 | 45,262 (28,635-89,909) | 110 (80 – 190) | 6 (5 – 8) | 3,577 | 2,201 |
|  | Langerhans cells | 4 | 12,719 (10,109-13,531) | 380 (350 – 480) | 5 (5 – 5) | 3,502 | 2,282 |
| **Peritoneal dialysate** | Macrophage | 5 | 678,691 (81,411-1,823,865) | 70 (30 – 100) | 64 (8 – 75) | 3,590 | 2,288 |
|  | myDC | 4 | 77,961 (10,153-200,000) | 110 (90 – 460) | 9 (5 – 19) | 3,452 | 2,351 |
| **Colon** | Macrophage (normal colon) | 5 | 12,673 (6,019-36,312) | 340 (160 – 1,590) | 5 (4 – 10) | 3,472 | 2,174 |
|  | Macrophage (CRC) | 7 | 22,645 (1,794 – 100,530) | 192 (41 -1,570) | 4 (2 – 6) | NA | NA |
|  | Normal colon (bulk) | 2 | 100,000; 550,000 | 108; 86 | 11, 47 | 4,148 | 3,184 |
|  | CRC (bulk) | 2 | 940,000; 6,000,000 | 207; 33 | 194, 199 | 3,883 | 3,883 |
|  | Normal epithelial CD166^het^ CD44^-^ | 7 | 530,624 (225,760-1,284,083) | 43 (9 – 72) | 16 (12 – 25) | 4,490 | 2,794 |
|  | Normal epithelial CD166^+^ CD44^+^ | 5 | 26,836 (17,556-108,404) | 164 (88 – 268) | 5 (4 – 10) | 4,417 | 2,976 |
|  | CRC epithelial CD166^het^ CD44^-^ | 7 | 202,439 (82,518-579,438) | 44 (19 – 497) | 13 (4 – 41) | 4,413 | 3,019 |
|  | CRC epithelial CD166^+^ CD44^+^ | 6 | 9,536 (410-22,724) | 343 (211 – 5,122) | 2.9 (2 – 9) | 4,320 | 2,881 |
| ***In vitro* cell models** | cMo unstimulated | 7 | 30,000 (30,000 – 30,000) | 123 (113 – 143) | 4 (3 – 4) | NA | NA |
|  | cMo LPS stimulated | 7 | 30,000 (30,000 – 60,000) | 117 (60 – 147) | 4 (3 – 5) | NA | NA |
|  | cMo R848 stimulated | 6 | 30,000 (30,000 – 30,000) | 120 (103 – 140) | 4 (3 – 4) | NA | NA |
|  | myDC unstimulated | 7 | 30,000 (30,000 – 30,000) | 130 (113 – 197) | 4 (3 – 6) | NA | NA |
|  | myDC LPS stimulated | 7 | 30,000 (30,000 – 30,000) | 123 (103 – 207) | 4 (3 – 6) | NA | NA |
|  | myDC R848 stimulated | 7 | 30,000 (30,000 – 30,000) | 120 (103 – 190) | 4 (3 – 6) | NA | NA |
|  | pDC unstimulated | 6 | 60,000 (30,000 – 60,000) | 65 (47 – 157) | 4 (3 – 5) | NA | NA |
|  | pDC CpG stimulated | 6 | 30,000 (30,000 – 60,000) | 112 (60 – 140) | 4 (3 – 4) | NA | NA |
|  | pDC R848 stimulated | 6 | 30,000 (30,000 – 60,000) | 98 (62 – 153) | 4 (3 – 5) | NA | NA |
|  | MD MAC unstimulated | 3 | 240,400 (98,200 – 535,000) | 226 (139 – 354) | 54 (35 – 74) | NA | NA |
|  | MD MAC M1 (IFNγ) | 3 | 261,400 (136,400 – 813,000) | 288 (125 – 369) | 75 (50 – 102) | NA | NA |
|  | MD MAC M2 (IL4) | 3 | 448,000 (65,500 – 796,800) | 185 (84 – 591) | 67 (39 – 83) | NA | NA |

*cMo,* classical monocyte; *iMo,* intermediate monocyte; *myDC,* myeloid dendritic cell; *pDC,* plasmacytoid dendritic cell; *CRC,* colorectal cancer; *MD MAC,* monocyte-derived macrophage; *NA*, data not available.
